# Dedicated core-on-anvil production of bladelet-like flakes in the Acheulean at Thomas Quarry I - L1 (Casablanca, Morocco)

**DOI:** 10.1038/s41598-020-65903-3

**Published:** 2020-06-08

**Authors:** Rosalia Gallotti, Abderrahim Mohib, Paul Fernandes, Mohssine El Graoui, David Lefèvre, Jean-Paul Raynal

**Affiliations:** 1 Université Paul Valéry Montpellier 3, CNRS, LabEx Archimede - ANR-11-LABX-0032-01 - and UMR 5140 Archéologie des sociétés méditerranéennes, Campus Saint Charles, 34199 Montpellier, France; 2 Université Bordeaux 1 UMR 5199 PACEA-PPP, Bâtiment B18 allée Geoffroy Saint-Hilaire CS 50023F, 33615 Pessac, Cedex France; 3 Centre d’interprétation du Patrimoine du Gharb, Direction provinciale de la Culture, Quartier administratif, Bd Mohamed V, Kenitra, Morocco; 4 SARL Paleotime, 6173 rue Jean Séraphin Achard Picard, 38250 Villard-de-Lans, France; 5 Institut National des Sciences de l’Archéologie et du Patrimoine, Madinat Al Irfane, Angle rue N°5 et rue N°7, Rabat-Institut, BP, 6828 Rabat, Morocco; 6 Department of Human Evolution, Max Planck Institute for Evolutionary Anthropology, Deutscher Platz 6, 04103 Leipzig, Germany

**Keywords:** Archaeology, Evolution, Anthropology, Archaeology

## Abstract

The ability to produce large cutting tools (LCTs) is considered as the technological marker of the Acheulean and the indicator of a greater technological complexity compared to the previous Oldowan. Although Acheulean techno-complexes are also composed of a concurrent core-and-flake technology, the iconic handaxes have attracted more attention than any other lithic component. Consequently, little is known of the small and medium-sized flake productions (small flaking), especially starting from 1 Ma, when handaxe and cleaver manufacture becomes intensive and widespread across Africa, including the Atlantic coastal regions of Morocco. Research at Thomas Quarry I yielded a rich early Acheulean lithic assemblage, mainly composed of quartzite LCTs and small flaking, together with a small-sized flint production. Here, we report a particular aspect of this flint assemblage, i.e. a flint bladelet-like flake production. This process represents a discrete technical behaviour among those related to small flaking both in quartzite and flint: pebbles were flaked using the bipolar-on-anvil technique repeatedly employing a specific method to produce bladelet-like flakes. This production represents the oldest dated occurrence of bladelet-like technology in Africa and reveals technical competencies hitherto unknown for these periods, providing further elements for the techno-economic diversification of the African Acheulean.

## Introduction

Large shaped tools (length or width ≥ 10 cm), made on large flakes, cobbles, or tabular clast blanks, are the hallmark of the African Acheulean from its emergence at ~1.8 Ma and for the subsequent 1.5 million years^1–22^. From the first definition of the term Acheulean^23^, although the techno-complexes are also composed of a concurrent core-and-flake technology, only handaxes and cleavers were used for grouping together different lithic assemblages scattered over space and time under this label^18,19,24^. Their degree of refinement was adopted as a parameter for defining Acheulean technological development and variability^1,2,5,6,14^ and the ability to shape macro-tools was equated to a more complex behaviour than the Oldowan core-and-flake based technology^22,25,26^.

In recent years, research focused on small-medium sized flake productions in the early East African Acheulean has modified previously established paradigms identifying the main innovations that distinguish the early Acheulean from the Oldowan technology, partially superseding the handaxe focus in Acheulean studies^8,11,15,17,27–32^. Unfortunately, the study of large tools clearly overshadows occurrences of smaller artefacts among African late Early/early Middle Pleistocene Acheulean assemblages. Little is known of these tool kits, when macro-tool productions become intensive and standardized^19^, and the relevance of the small flaking to hominin behavioural variability remains to be investigated.

Here, we present a bladelet-like flake production identified in the late Early Pleistocene African Acheulean of Thomas Quarry I (ThI-L1) at Casablanca (Morocco). This production was achieved through a techno-economic process never documented in the African Acheulean. A set of flint cores and flakes display a specific technical process for a recurrent bladelet-like flake production, flaking pebbles through the bipolar-on-anvil technique. Although this technique is the best solution to exploit very small clasts^33,34^, in this case the core convexity management and maintenance show a more complex know-how intentionally involved for detaching as many as possible bladelet-like products. This process is independent from the rest of the flint artefacts focused on the small flake production and it has not been identified within the quartzite small flaking. Furthermore, this is the only known case in the African Acheulean of a bladelet-like production recorded with LCT manufacture in the same archaeological layer^35–37^.

## THI–L1 context

**Figure 1:**
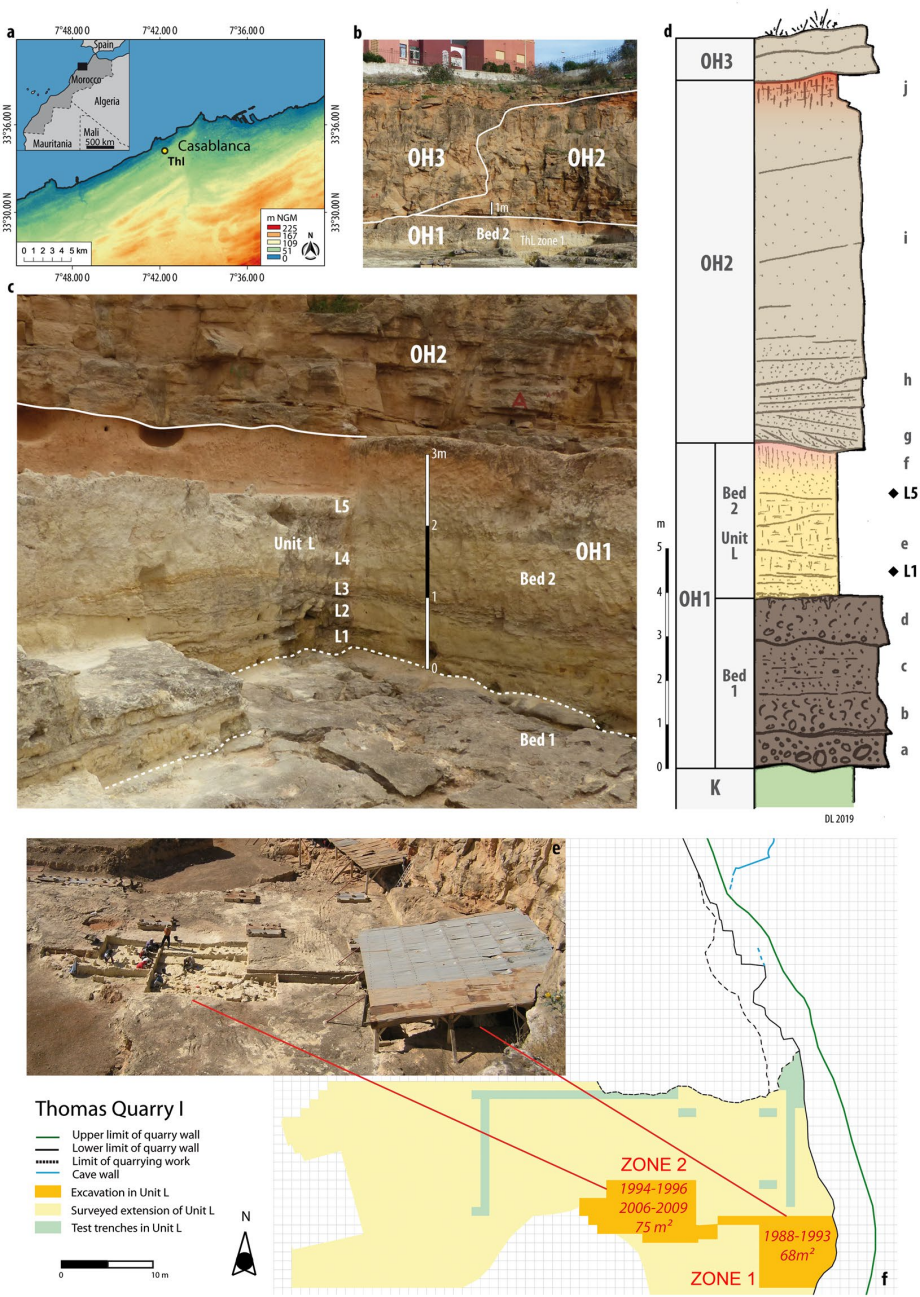
(**a**) Location of ThI (drawing by M. Rué). (**b**) ThI: the Oulald Hamida Formation Members OH1 to OH3 (photo and drawing by D. Lefèvre 2014). (**c**) ThI-L1, Zone 1: OH1 Bed 2 - Unit L deposits (photo and drawing by D. Lefèvre 2016). (**d**) ThI: stratigraphy of the OH1 and OH2 Members (drawing by D. Lefèvre 2019). OH1 Member*:* unconformity above the Cretaceous limestone (K); Bed 1: (a) coarse calcirudite; (b) coarse coquinoid biocalcarenite, (c) coarse biocalcarenite and (d) coquinoide calcarenite; Bed 2- Unit L: (e) large scale trough cross-bedded fine to coarse sands and calcareous mudstone banks. L1 and L5 archaeological layers; (f) bioturbated and decarbonated bioclastic aeolian sands. OH2 Member: erosional surface boundary; (g) curved cross-bedded coarse biocalcarenites; (h) finer inclined planar-bedded biocalcarenites; (i) massive banks of aeolianites; (j) fersialsol pedogenesis at the top. (**e**) ThI-L1 during 2007 excavation (photo by J.-P. Raynal 2007); (**f**) map of ThI with extension of unit L and location of the excavation zones (drawing by R. Gallotti).

The Casablanca region is well known for its exceptional development of Quaternary littoral deposits, beginning in the Upper Miocene and spreading over the Plio-Quaternary times with an extremely detailed registration of the global climatic cycles^38–42^. Construction works that started at the dawn of the twentieth century in the city of Casablanca demanded the opening of large quarries and incidentally revealed the longest Acheulean sequence in North Africa recorded in an indisputable stratigraphic context^37,43^. One of these quarries, Thomas Quarry I (ThI; Fig. 1a), was made famous in 1969 by the discovery of a human half-mandible in the *Grotte à Hominidés*^44^. In 1985, ThI was re-examined revealing the presence of the earliest North African Acheulean in the lower unit L^45^.

Unit L corresponds to the Bed 2 of the Oulad Hamida Formation Member 1 (Fig. 1b-d). It is a 2–3 m succession of yellow lenticular limestone beds with a cross-bedded architecture, deposited in a littoral fluvio-lacustrine hydrosystem with shifting channels and a temporary water table, followed by pedogenised aeolian sands^40–42^. Archaeological layers are distributed on temporarily exposed surfaces at several levels of the sedimentary piling. Unit L was dated using the OSL signal of quartz grains to between 0.8 and 1.2 Ma^46^, with large uncertainties. However, the detailed analysis of the complete lithostratigraphy of the Casablanca sequence demonstrates that OHF Member 1 lays below formations representing three highstand sea-levels older than MIS 15, probably MIS 17 to 21 if the record is complete, more if it is not, pushing it back to 1 Ma at least in the Early Pleistocene^41,42^ (see Supplementary Information text and Supplementary Fig. S1).

Faunal remains are rare and consist mostly of hippopotamus, with some *Elephas* and *Equus*. A *Kolpochoerus* tooth probably belongs to *K. maroccanus*, in agreement with an Early Pleistocene age. Only a few rodent teeth have been recovered. *Ellobius*, a genus that appears at Tighenif, is absent; this might merely be absence of evidence, but both the *Paraethomys* and *Gerbillus* differ from those found in later units of the quarry, suggesting a significant age difference^47^. Bone surfaces are usually unreadable, preventing a detailed taphonomic analysis.

In Bed 2, the archaeology is limited to archaeo-stratigraphic sub-units L1 at the base and L5 at the top. L1 has been systematically excavated (1988–1996 and 2006–2009) on two areas (Zone 1 of 68 m^2^ and Zone 2 of 75m^2^) and several test trenches (Fig. 1e,f).

L1 yielded a rich artefact assemblage of quartzite and flint together with unmodified cobbles/pebbles and few faunal remains. In this work we only take into consideration the lithic assemblage belonging to the excavation Zone 2, composed of 2973 artefacts and 3109 unmodified lithic items, stored in the field laboratory at ThI (Table 1). Here, we briefly present the lithic assemblage composition, a summary of the quartzite knapping activities, and we focus on a detailed description of the flint industry, especially of the bladelet-like flake production.

**Table 1:** Components of the lithic assemblage of ThI-L1, Zone 2.

Components	Quartzite		Flint	
	N	%	N	%
Cobbles and pebbles with percussion marks	28	0.6	6	0.6
Cores	212	4.2	107	10.4
Core fragments	42	0.8	28	2.7
Flakes	525	10.4	175	16.9
Bladelet/like flakes	—	—	35	3.4
Broken flakes	648	12.8	46	4.4
Retouched flakes	2	0.0	3	0.3
Large flakes	12	0.2	—	—
LCTs	110	2.2	—	—
LCT points	10	0.2	—	—
Wastes	912	18.1	72	7.0
***Total artefacts***	***2501***	***49.5***	***472***	***45.7***
Cobbles	170	3.4	—	—
Broken cobbles	22	0.4	—	—
Pebbles	2324	46.0	534	51.6
Broken pebbles	14	0.3	28	2.7
Natural fragments	17	0.4	—	—
***Total unmodified items***	***2547***	***50.5***	***562***	***54.3***
***TOTAL***	***5048***	***100.0***	***1034***	***100.0***

## Lithic Assemblage Composition

The ThI-L1 lithic assemblage of Zone 2 is composed of quartzite, which dominate numerically (81.9% of the unmodified items and 84.1% of the artefacts), and flint (18.1% of the unmodified items and 15.9% of the artefacts). Flint derived from the phosphatic plateau in the hinterland of the Meseta and is available in secondary deposits near the site. Quartzites are abundantly available in local primary and secondary sources.

**Figure 2:**
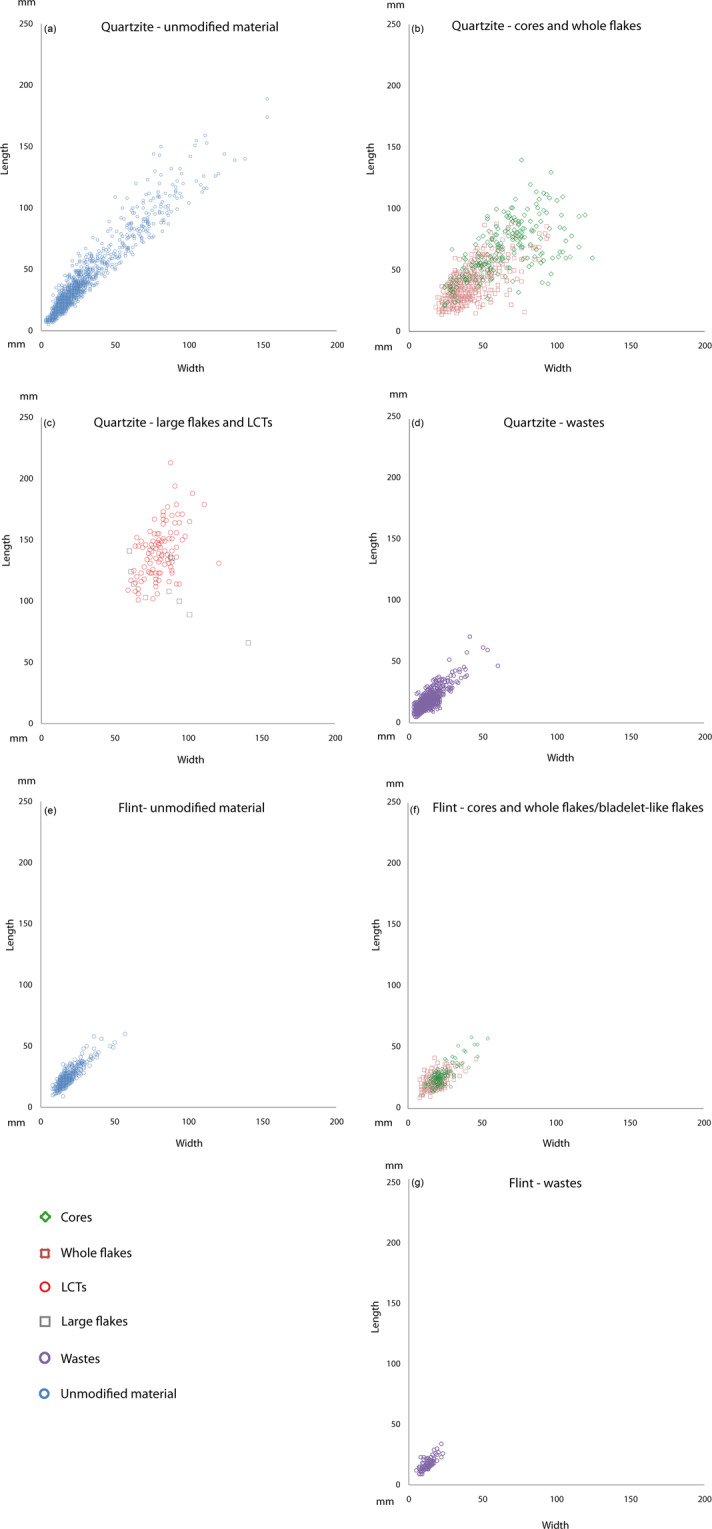
Size distribution (mm) of quartzite unmodified material (**a**), quartzite cores and whole flakes (**b**), quartzite large flakes and LCTs (**c**), quartzite wastes (**d**), flint unmodified material (**e**), flint cores and whole flakes/bladelet like-flakes (**f**), and flint wastes (**g**).

Unmodified quartzite and flint material recorded in L1 layer is mainly composed of pebbles and cobbles (Table 1; Fig. 2a,e), which represent both material accumulated by natural agents and manuports as a potential source of raw material for knappers. Some small quartzite and flint pebbles are present at bottom of water assisted deposits of Bed 2 sub-unit L1. This sedimentary architecture implies that low current transported and deposited sediments. Fabric analysis of large elongated quartzite artefacts and bones distributed on the archaeological surfaces attests of their slight re-orientation^48^. Nevertheless, large quartzite cobbles and implements were not water deposited, preserving cutting edges as fresh as the flint artefacts, though small pebbles and fragments may have been displaced. However, observation made during the excavation and states of preservation of both quartzite and flint objects do not bring any argument in favour of a residual deposit cumulating different occupational sub-units eroded down to a single layer. Some hippopotamus remains found in layer L1 could suggest that flooded archaeological surfaces may have been disturbed by these animals. Detailed observation of the flint artefact and pebble cortex reveals a long history in marine environment, which is not erased by fluvial transportation. This means that either pebbles derive from marine deposits very close to their final deposition place (THI-L1 deposit) or that humans collected them directly in marine beaches or in slightly derived deposits (for a more detailed description of the mineral resources exploited by hominins at ThI-L1 see the Supplementary Information text and Supplementary Figs S2 to S4).

The size distribution of flint and quartzite artefacts reflects that of the unmodified pebbles and cobbles recovered in the archaeological deposit (Fig. 2). Two main quartzite artefact production systems coexisted at ThI-L1: one is focused on the production of small to medium-sized flakes, the other is devoted to the manufacture of LCTs (Table 1). LCTs (picks, cleavers, bifacial and trihedral tools) are mainly made on large cobbles. Large flakes from cobbles and boulders used as LCT blanks are rather rare. The quartzite assemblage also contains numerous cores and small-medium sized flakes (Table 1)^36,37^. Several flaking methods (unifacial unidirectional, bifacial partial, peripheral unidirectional, multifacial multidirectional and discoid) mainly exploited medium-sized cobbles to produce small-medium sized flakes. Small quartzite cores (25–50 mm) usually correspond to an overexploitation of the cobble blanks through a multifacial multidirectional method. Flint was exclusively exploited for small flaking.

The number of flakes (whole, broken, and retouched flakes) does not fit with the number of the negative scars that can be observed on the whole cores. Additionally, some cores have been intensively exploited and some flakes exhibit more than one negative scar on the dorsal face, which suggests that the deficit of flakes is larger than we can estimate based on the negative scar count only. This deficit increases if we also consider the shaping of the LCTs. In any case, flakes represent all the flaking stages and methods identified in core analysis. Thus, the large deficit of flakes does not document a spatial and temporal segmentation of the *chaînes opératoires* and the presence of very small lithic elements (<10 mm; Fig. 2a,b,d-g) does not point to a winnowing by natural agents. We have to consider in this matter that at Thomas Quarry I Bed 2 deposits outcrop on a thousand square meters (Fig. 1f). This surface only represents a fraction of the Bed 2 which extends on several hundred meters to the South-West and is fossiliferous all along. In such an extended open-air site, organized on the edge of a fluctuating water body, we must expect a fragmentation of tasks and mobility of artefacts within it, along with disturbance caused by temporary flooding. Thus, counts of the different knapping products belonging to the excavations may not be representative of the site composition and limits discussion of the actual *chaîne opératoire* segmentation.

## Flint Knapping Activities

The flint assemblage is composed of 472 artefacts (2591 g) and 562 unmodified items (3492 g) (Table 1). The flaked flint assemblage was exclusively produced from pebbles (30 to 60 mm). Flint pebbles do not have angles generated by the intersection of two or more surfaces that can facilitate the first production phases. They are rounded with mainly ovoid and subcircular shapes and bi-convex, plano-convex, and flat cross-sections (Supplementary Figs S5, S6).

### Small flake cores

Small flake production is documented by 68 cores exploited by simple (n = 29; one or two removal scars without obvious organization^49^) and organized flaking (n = 39) performed using the free-hand percussion with hard hammerstones. Other seven cores have been exploited through the bipolar-on-anvil longitudinal exploitation.

**Figure 3:**
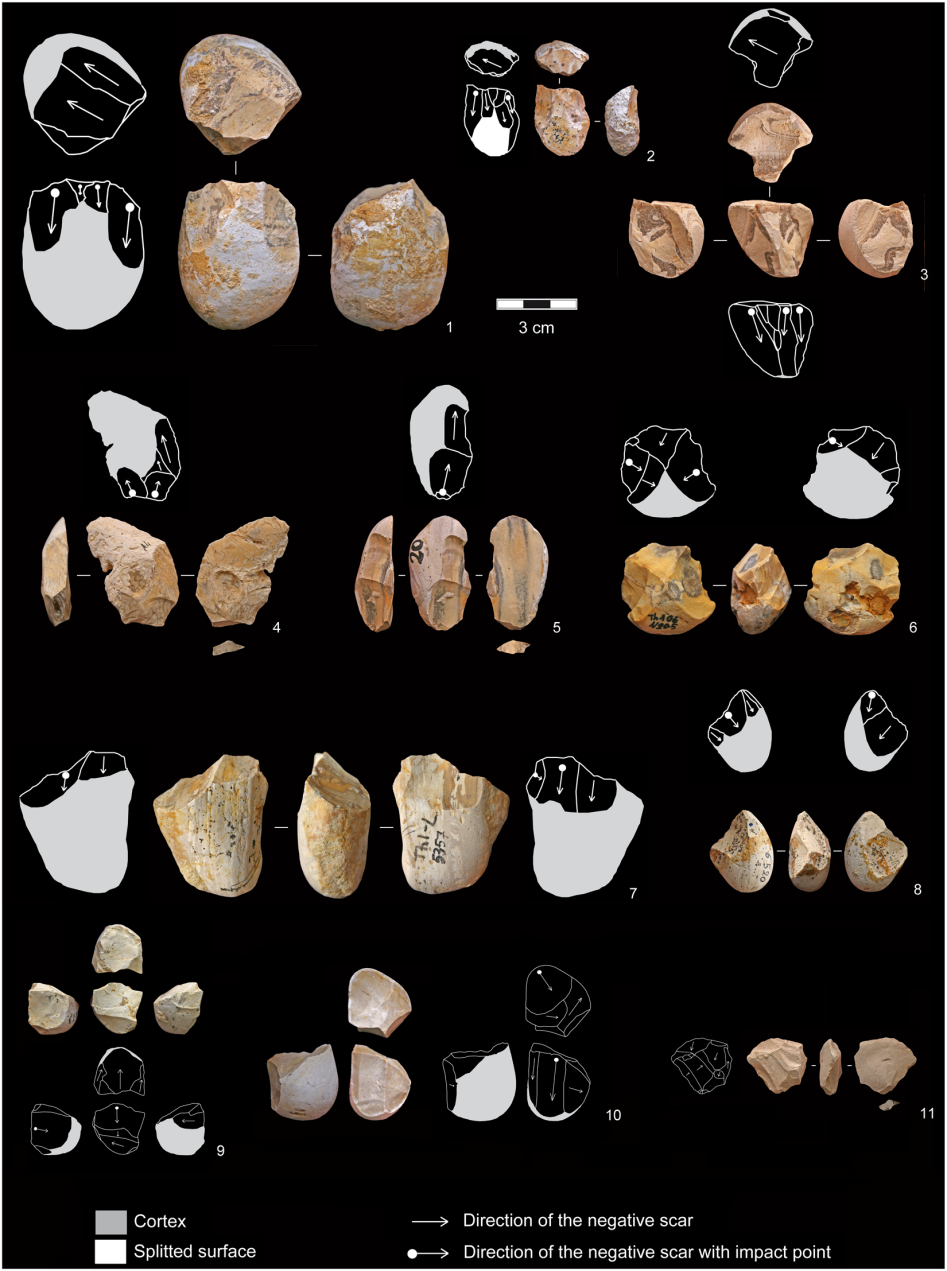
Flint freehand exploitations. 1–3: unifacial unidirectional cores with rectified striking platform; 4,5: flakes with unidirectional negative scars on the dorsal face; 6–8: bifacial partial alternating cores; 9,10: multifacial multidirectional orthogonal cores; 11: hinged flake with multidirectional negative scars on the dorsal face (photos and drawings by R. Gallotti).
- bifacial partial alternating (n = 12). Cores exhibit removals on two adjacent surfaces, and each negative scar is used alternatively as a striking platform to flake the adjacent plane. The blanks are bi-convex ovoid pebbles, systematically exploited on the transversal axis (Fig. 3: 7,8). Only one core shows a semi-peripheral exploitation (Fig. 3: 6). Angles between the two flaking surfaces vary from 45° to 70°.
- multifacial multidirectional orthogonal (n = 10). These cores are overexploited (five to seven flakes on three to four surfaces) and smaller than the previous ones (Fig. 4a). Core surfaces were alternatively flaked through multidirectional removals respecting the orthogonal angles among flaking surfaces. No specific platform rectification was conducted insofar as each negative served as a striking platform for the following removal on a secant and orthogonal face (Fig. 3: 9,10).

Among the cores flaked using a freehand percussion and organized exploitation, three flaking methods have been identified:

- unifacial unidirectional (n = 17). Core blanks are mainly ovoid bi-convex and plano-convex pebbles, rarely subcircular bi-convex. The flaked surface usually corresponds to the longest natural face of the pebble, exploited to produce one to two series of three to seven elongated flakes from a striking platform rectified by one or two removals to create a suitable angle between 62° and 89° (Fig. 3: 1–3).

**Figure 4:**
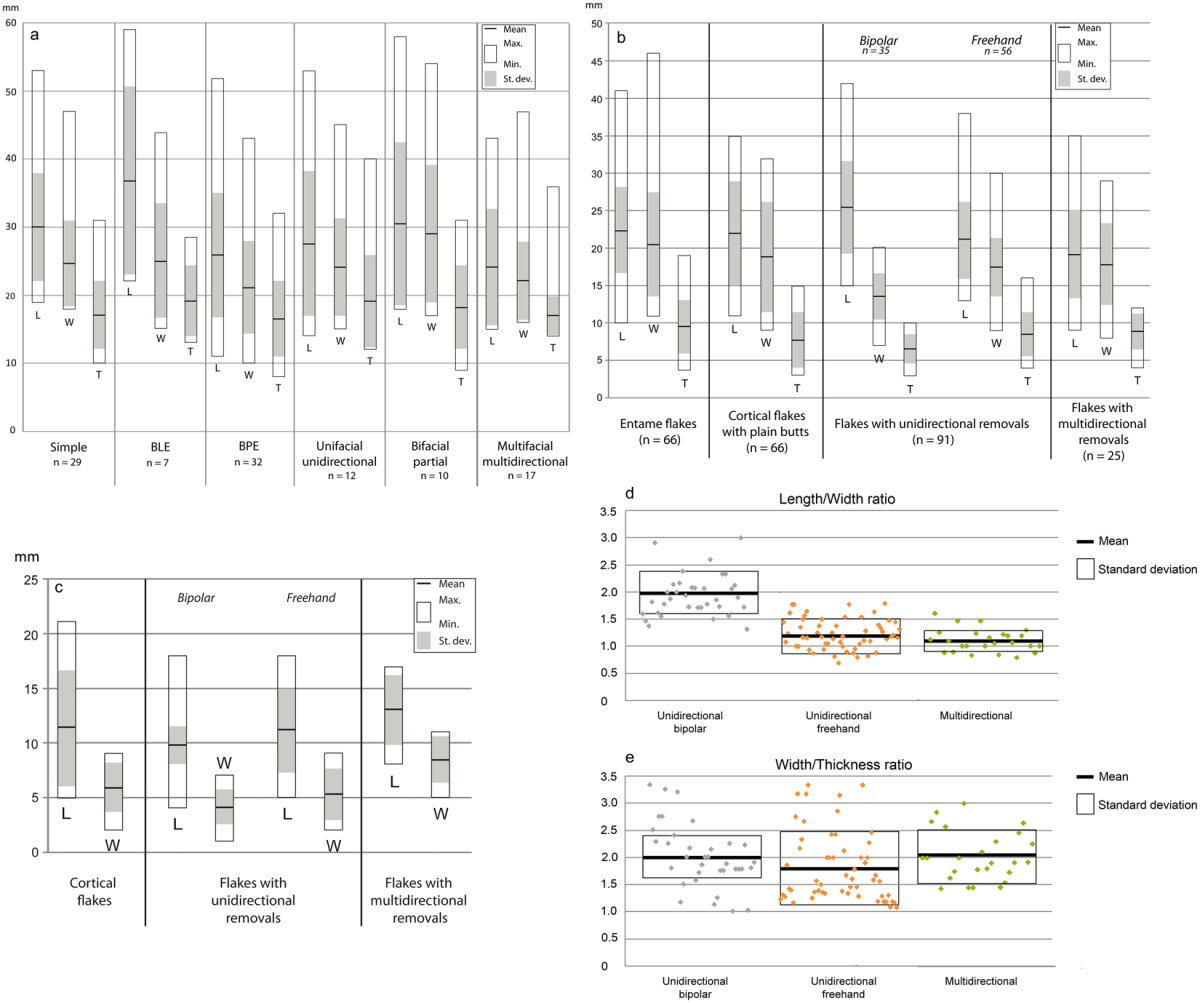
Dimensional distribution of flint cores and flakes. (**a**) Length (L), width (W), and thickness (T) distribution (mm) of the cores that belong to flint flaking, grouped by flaking method; (**b**) length (L), width (W), and thickness (T) distribution (mm) of the whole flakes; (**c**) length (L), and width (W) distribution (mm) of the whole flake butts; (**d**) log flake length to width ratio; (**e**) log flake width to thickness ratio. BLE: bipolar-on-anvil longitudinal exploitation. BPE: bipolar-on-anvil semi-peripheral exploitation.

**Figure 5:**
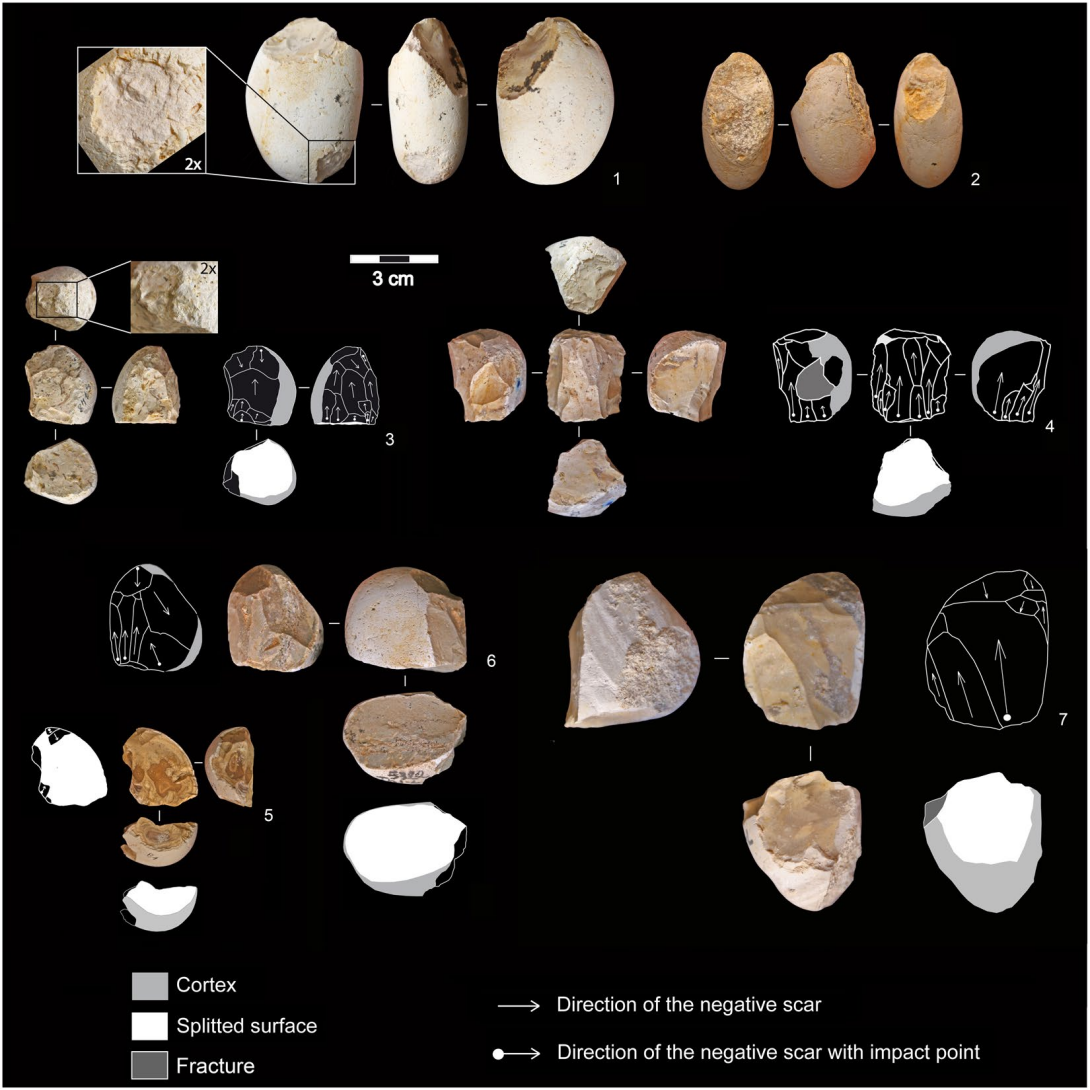
Flint cores belonging to bipolar-on-anvil flaking. 1,2: BLE cores showing two negative scars generated along the longitudinal axis from the pebble extremity in contact with the anvil. The opposite cortical extremity displays marks of percussion (n.1). 3–7: BPE cores showing a split surface along the transversal axis of the pebble and elongated peripheral negative scars. Percussion marks on the opposite cortical side are visible in core n. 3. Core n. 5 shows a split fracture along the longitudinal axis (photos and drawings by R. Gallotti). BLE: bipolar-on-anvil longitudinal exploitation. BPE: bipolar-on-anvil semi-peripheral exploitation.

The bipolar-on-anvil longitudinal exploitation (BLE) concerns flint bi-convex and plano-convex ovoid pebbles (n = 7). The bipolar percussion simultaneously yielded two flakes generated along the longitudinal axis from the pebble extremity in contact with the anvil (Fig. 5: 1,2), given the rebound force generated by the anvil’s resistance to the force of the hammerstone. The other pebble extremity, located along the same axis, shows the percussion impact damages produced by the hammerstone’s action (Fig. 5: 1). Some of the percussion marks identified on few pebbles could correspond to an attempt of splitting along the longitudinal axis (Supplementary Information text and Supplementary Fig. S8).

### Bladelet-like flake cores

**Figure 6:**
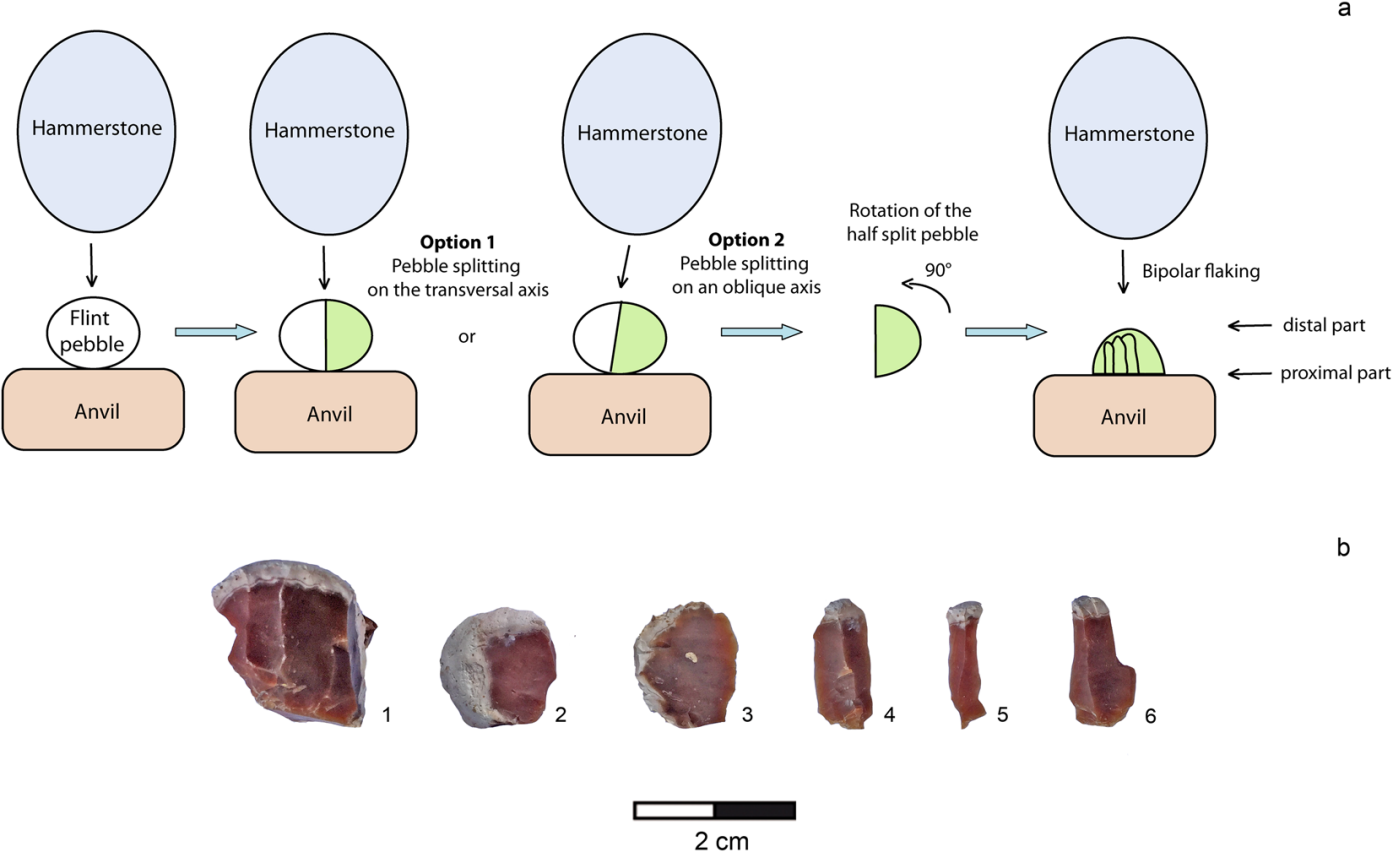
(**a**) Operational scheme of the BPE exploitation (drawing R. Gallotti); (**b**) experimental BPE exploitation (1: core; 2,3: flakes belonging to the first flaking phase; 4–5: bladelet-like flakes) (photo by A. Mohib). BPE: bipolar-on-anvil semi-peripheral exploitation.

Bladelet-like flake production has been identified mainly thanks to the analysis of 32 flint cores showing a specific process, the bipolar-on-anvil semi-peripheral exploitation (BPE), independent from the other flint flaking methods and absent in the quartzite flaking (Fig. 6a). These cores show one horizontal or slightly oblique split surface along the transversal axis of the pebble and percussion marks on the opposite side (Fig. 5: 3,4). The battered area could be associated to one small negative scar which seems due to the percussion rather than to an intentional rectification of the striking platform (Fig. 5: 4). The semi-peripheral flaking surface displays several negatives of bladelet-like flakes, most of them with a rippled surface, whose impact point generated from the split surface. In some cases (n = 6), cores exhibit small negative scars with an opposite direction (Fig. 5: 3,6,7). The edge of the split surface shows micro-fractures and micro-detachments which overlap one to each other. These cores are overexploited as documented by the high number of the negative scars varying from five to 10 distributed in one to three series. They document attention to the semi-peripheral longitudinal convexity during flaking, which allows the detachment of a high number of bladelet-like flakes (always relative to the small dimensions of the blank).When the blank morphology can be recognized after the residual cortical part, we observe that the convexity management is favoured by the choice of ovoid biconvex and thick pebbles as core blanks. The choice of the blank morphology is fundamental in the convexity management, because it is maintained mainly through core rotation. Flake negatives are visible on the cores: they are related to the first phase of the flaking and/or they are due to a failed attempt of convexity maintenance.

In order to understand the role of the pebble split surface and the core positioning on the anvil, we performed experimentation to reproduce this type of core reduction. Our experimental replication demonstrates that these bipolar cores derive from an indirect fracture technique that follows the pebble splitting along the transversal axis. When a pebble is split, the fragments can be hemispheric or plano-convex. The flat surface is appropriate to stabilize the half-pebble on the anvil (proximal portion) and strike the convex opposite surface (distal portion) with the hammerstone. According to the archaeological core exploitation patterns, the position of the striking surface (distal portion) and the split surface resting on the anvil stabilizing the core (proximal portion) remains stable during pebble exploitation (Fig. 6). Core is rotated according to the longitudinal axis to exploit its periphery and no orthogonal rotation of the core is operated. Detachments usually occur one at a time, rarely in multiples. Micro-fractures and micro-flake scars are generated by the proximal rebound force along the edge of the split surface in contact with the anvil. A large quartzite hammerstone has been used to split the pebble along the transversal axis, while a smaller hammerstone on flint or quartzite has been used for flaking so that an excessive force does not split the plano-convex core in half. Nevertheless, it cannot be excluded that a split fracture along the longitudinal axis can intervene when the core is overexploited, as demonstrated by two archaeological cores (Fig. 5: 5).

The dimensions of these bipolar cores do not differ significantly from those of the cores flaked by freehand technique (Fig. 4a). However, their overexploitation coupled with the fact that these cores are flaked on half-pebbles suggest that larger pebbles were selected by the knappers to operate the transversal split.

### Flakes and bladelet-like flakes

A total of 259 flakes (175 whole, 35 bladelet-like, 46 broken, and three retouched) belong to the flaking of flint pebbles (Table 1). Thirteen whole flakes <2 cm with a plain butt display a dorsal face created by a large negative scar whose direction is impossible to recognize. Accordingly, these flakes are counted in waste category including the flake fragments that cannot be situated in the *chaîne opératoire*^50^.

The proportion of flakes bearing cortex is high (n = 180; 69.5% of the flakes) since, even in the case of overexploited cores, the small size of the flaked pebbles hardly allows the production of flakes that do not bear residual cortical portions.

**Figure 7:**
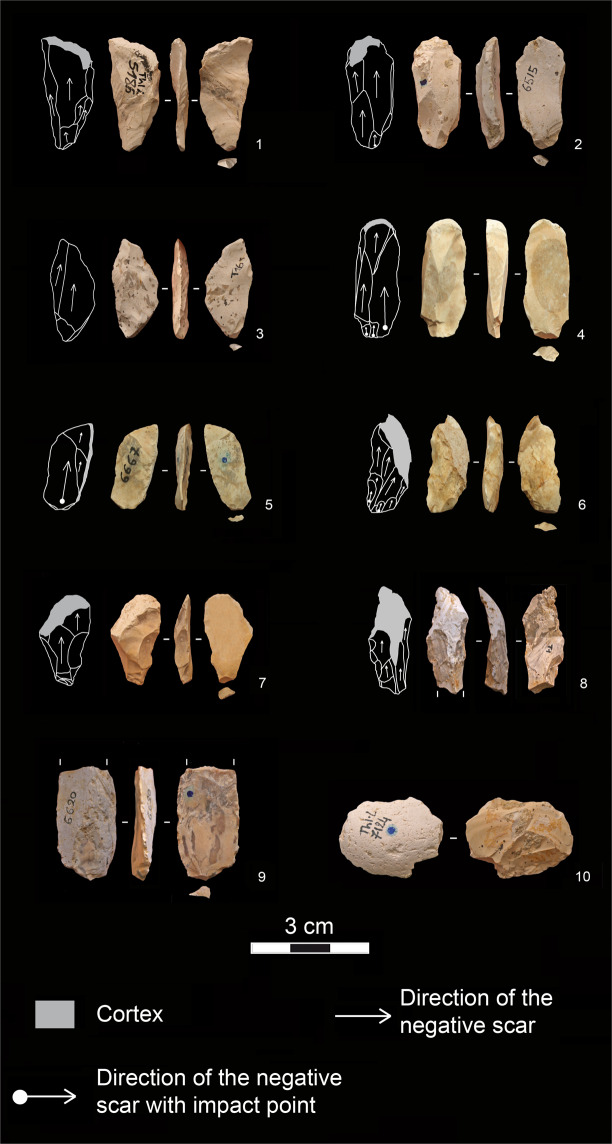
Products of the bipolar-on-anvil semi-peripheral exploitation (BPE). 1–7: bladelet-like flakes; 8, 9: broken bladelet-like flakes; 10: *entame* flake (photos and drawings by R. Gallotti).

Sixty-six *entame* flakes are present in the flint assemblage (31.4% of the whole flakes). Five of them display some technical traits of the bipolar-on-anvil percussion such as sheared or crushed bulbs and butts as well as rippled ventral surfaces and a cracked outline (Fig. 7: 10). They are mostly subquadrangular and subcircular in shape and only a few are elongated. Their dimensions do not substantially differ from those of the other whole flakes (Fig. 4b).

Fifteen flakes are completely cortical with a plain butt (7.1% of the whole flakes). The flaking angle (interior platform angle) is comprised between 90° and 121°. No indisputable traits of the bipolar-on-anvil technique have been recognized. Their dimensions are very similar to those of the *entame* flakes (Fig. 4b).

Flakes with a unidirectional negative scar pattern on the dorsal face, parallel to the flaking axis, with frequent cortical edge(s) constitute a large set (n = 91; 43.3% of the whole flakes). Thirty-five of them show two to six negative scars and bear traits of the bipolar-on-anvil percussion and notably:

- hackles on the ventral face and on the negative scars on the dorsal face (a fracture mark, which develops perpendicular to a fracture front, and therefore spreads radially from the impact point; Fig. 7: 1,3);
- a rippled ventral face (Fig. 7: 1, 3–5);
- battering marks on the cortex distal end when present (n = 15), corresponding to the percussion of the distal part of the core (Fig. 7: 2,4,7,8), especially visible in the case of plunging flakes (n = 7; Fig. 7: 1,8);
- a cracked outline mainly in the case of cortical edges (Fig. 7: 1, 6–8,9);
- frequent bulb scars (Fig. 7: 2,3);
- crushing and splintering of the proximal part of the dorsal face adjacent to the butt (Fig. 7: 4,7);
- crushed (n = 8) or plain (n = 27) butts. Plain butts are slightly narrower and thinner than those of freehand flakes with the same dorsal pattern (Fig. 4c). The flaking angles varies between 92° and 116° and match the angles between the flat split surface of the core laid on the anvil and the flaking surface. Bipolar flakes are more elongated than the freehand unidirectional flakes and other whole flakes with a multidirectional flake scar system (Fig. 4b). The mean ratio of length to width is 1.94, a value that points to a bladelet-like flake production (Fig. 4d). Besides, bipolar flakes have slightly thinner cross-sections (average W:T ratio = 2.02) than freehand flakes do (average W:T ratio = 1.85; Fig. 4e).

Some technical traits related to the bipolar-on-anvil percussion are recognizable on 38 of the 46 broken flakes (Fig. 7: 8,9), although their identification must be taken with caution given the lack of a portion of the flake. However, bipolar-on-anvil percussion usually produces more fragments than freehand percussion, simply because of breakage of immobilised flakes^51^.

The remaining 56 flakes bearing unidirectional scar pattern on the dorsal face (one to four removals) belong to the freehand percussion. They are smaller than the bipolar ones (Fig. 4b) and less elongated (average L:W ratio = 1.23; Fig. 4d). 78% of them retain cortex on the distal-lateral portion. The high percentage of flakes with residual cortex is probably due to the limited exploitation of the unifacial unidirectional and bifacial partial cores from which they could derive. Plain butts are present on 38 flakes, with flaking angles between 95° and 126°. Usually, plain butts are present on elongated flakes, according to the unifacial unidirectional exploitation (Fig. 3:4,5). The smaller flakes, generally with sub-quadrangular or sub-circular shapes, present mainly cortical or cortical/flat butts with obtuse flaking angles (114° to 127°), probably because they belong to the bifacial partial exploitation.

Flakes with a multidirectional scar pattern on the dorsal face (n = 25; 11.9% of the whole flakes) do not show technical traits of the bipolar-on-anvil technique. Moreover, no rotation of the core in the bipolar-on-anvil exploitation has been identified. They are frequently subquadrangular and smaller than the flakes previously described (Fig. 4b). Eleven of them retain cortex on the distal and/or lateral portions and core edge flakes, implying core rotation. Negative scars, mainly orthogonal both with each other and with the flaking axis, range between two and eight and confirm the overexploited aspect of the multifacial multidirectional cores. Twelve flakes and most of the negative scars on the dorsal face are hinged removals (Fig. 3: 11). They have thicker and asymmetrical cross-sections (Fig. 4b) and longer and wider plain butts (Fig. 4c) with obtuse flaking angles (91°–105°).

Only three flint flakes have been retouched. The first two are cortical flakes with an abrupt retouch on the right lateral edge (Supplementary Information Fig. S7: 1,2); the third one is a cortical flake with a negative scar on the left side and a sub-parallel and invasive retouch on the distal edge (Supplementary Information Fig. S7: 3).

## Discussion

Knappers of ThI-L1 frequented a local lithospace rich in quartzites of all possible sizes abundantly available in primary and secondary sources, in which flint was present only as pebbles and in very small quantity^52^.

Large quartzite cobbles were turned into LCTs by shaping, whereas quartzite small and medium-sized cobbles were exploited to produce flakes. Knappers flaked also flint pebbles to produce small flakes, adopting some of the flaking methods documented for quartzite (unfacial unidirectional, bifacial partial, multifacial multidirectional) and mainly the free-hand technique.

Nevertheless, flint production at ThI-L1 shows also evidence of a specific technical process hitherto unknown for these periods: the intentional production of recurrent bladelet-like flakes through bipolar-on-anvil technique. Furthermore, this process is closely related to the use of flint and is absent in quartzite knapping activities, although knappers had a large quantity of pebbles available that could have been flaked according to the same process. Accordingly, the production of bladelet-like flakes exclusively of flint is most likely driven by specific needs, which for now remain unknown.

This process has wide implications on the current knowledge of the African Acheulean technical behaviors. The main novelty is the production of bladelet-like flakes itself in an Acheulean techno-complex with a strong macrolithic tendency. This production is not documented only by the mere presence of a few “laminar” small products, but is supported by the identification of an independent technical process (BPE) mainly based on the core analysis, that was achieved to detach products with specific metric features (i.e. length approximately twice than width) from a specific raw material. Another novelty is the exclusive use of an improved bipolar-on-anvil technique in two steps: 1) the splitting of the pebbles along the transversal axis to create a surface suitable to stabilize the core on the anvil, and 2) the extraction of bladelet-like products. Usually, the bipolar-on-anvil technique has been explained as a response to raw material constraints in order to maximally exploit small core blanks^53,54^ and sometimes considered as a technique used by less skilled knappers^55^. Recent experimentations to distinguish bipolar from free-hand technique in the early technologies show that bipolar knapping is conditioned by the blank morphology and produced shorter and thicker flakes with a high variability in shape and dimensions, and often involves core rotation usually orthogonal to the previous platform/flaking surface in order to pursuit flaking and to guarantee an intense exploitation^56,57^.

At ThI-L1, the adoption of a peculiar bipolar-on-anvil technique for a different process required more advanced competencies and skills and the respect of three requisites: 1) the application of the concept of anticipation in the choice of blanks (elongated pebbles suitable to be split along the transversal axis and with long peripheral convexities); 2) the opening of a surface laying on the anvil to ensure core stability during reduction; 3) an attempt to manage and maintain the natural peripheral convexity allowing a recurrent bladelet-like flake production.

For long time blade and bladelet productions were considered to be an indicator of distinctive cognitive capabilities mostly attributed to *Homo sapiens*^58,59^. Recent research demonstrates that this is clearly no longer the case^60,61^. A blade production dating to 545–509 ka has been discovered in the GnJh-42 and GnJh-50 Acheulean sites from the lower portion of the Kapthurin Formation (Kenya). Blades are of small dimensions and were detached through the free-hand technique. In these sites the production of LCTs is not documented and blade production has been interpreted as an increasing diversification of the Middle Pleistocene technical behaviours in East Africa which foreshadows the Acheulean-Middle Stone Age (MSA) transition^61^. A systematic and intense blade production is known in the Amudian assemblages of Qesem Cave (Israel) at 400–200 ka^62^. In Near East sequences, this is a locally new technology showing “planning and intensity not significantly different from Middle Paleolithic Mousterian industries, thus possibly reflecting a considerable change in human lithic technological behavior prior to the Middle Paleolithic period”^62^. Blade/bladelet productions are rare also at the late Middle/early Late Pleistocene. In North Africa, blades are present in the undated pre-Aurignacian of Libya^63^, in the early MSA in the valley of Egypt^64^, and in Aterian assemblages^65^. In sub-Saharan Africa also blade/bladelet production are present within certain assemblages attributed to the MSA^66–68^. However, blade/bladelet production becomes intensive and widespread only in later periods and is the hallmark of the Late Stone Age in Africa^69,70^. It is usually associated with microlith manufacture (small retouched or small backed geometric tools) and composite tool technology and considered as a “modern” technical behaviour^71^.

Blade/bladelet production since the Middle Pleistocene onwards is ruled by the predetermination of the products (i.e. the pre-planning of the metrical and morphological flake patterns before its detachment through one or more previous detachments^72^). Predetermination implies a “pre-planned débitage, organized in such away as to repeatedly produce blades or bladelets from a single core” and allows the production of standardized blade/bladelets, with parallel or nearly parallel edges^50^. This definition of pre-planning, formulated from the analysis of recent Palaeolithic assemblages, cannot be transferred to the bladelet-like flake production of THI-L1. The pre-planning in this specific Acheulean production dated to the end of the Early Pleistocene is achieved through a selection of the core blanks and the management of their geometrical features during flaking and not through the technical predetermination as defined above. Thus, the standardized morphological features of “classical” bladelets are absent at ThI-L1 and the identification of bladelet-like flakes rests on a length to width ratio that approximately equals 2. In a broader perspective, while the concept of predetermination appears for the production of LCTs from ~1 Ma^20,22^, it is not documented in the Acheulean small flaking, appearing with the Levallois concept from ~0.4–0.3 Ma onwards^37,73,74^.

Bladelet-like flake production of ThI-L1 seems to be episodic at the end of the Early Pleistocene and not followed by any further similar production for 0.5 Myr. The sporadic appearance and disappearance of technological innovations in Africa is not unusual^59^, as documented also in previous periods by for example the late Oldowan obsidian small points from Garba IVE^75^. Even the laminar production from the Kapturin Formation at ~0.5 Ma disappeared then reappeared 200,000 years later^61^. It has been argued that these occasional technical behaviours did not play a significant role in the hominin behavioral adaptations and in the evaluation of the Early/Middle Pleistocene technological complexity^61^. However, the complexity of a technical or adaptive behavior is difficult to establish in absolute terms, since the variables involved are multiple and diversified because strongly linked to different contexts, activities, and hominin species. The first appearance of new technical strategies, though sporadic as the bladelet-like flake production at ThI-L1, is important not because it indicates more complex cognitive skills by itself, but because it demonstrates hominins possessed technical know-how to create new solutions when need arose. Providing further data to the small flaking strategies, flint knapping from ThI-L1 well demonstrates that African Acheulean techno-economic strategies were much more flexible and diversified than the mere production of LCTs can document.

## Methods

Excavation of Th1-L was performed according to the stratigraphic sediment deposition. Spatial (x, y, z) data of all remains (worked and unworked lithics, as well as faunal remains) have been recorded. Sediments have been collected each 1 m^2^, dissociated with diluted acetic acid and sieved by water to recover lithic and faunal small fragments.

All knapped and unmodified lithic items have been analysed and measured. The study of the artefacts is founded on the technological approach, which is widely used in lithic analyses of most Early/Middle Pleistocene assemblages in East Africa, Europe, and Near East^4,8,10,11,15–18,20–22,24,27–32,49,75–78^. Following the *chaîne opératoire* concept^50,79–82^, we examined all of the technical sequences involved in lithic production as well as the related technical and cognitive competencies.

Unmodified items were classified according to their shape and size to appraise the blank geometry and dimensions in order to understand 1) if knappers operated specific choice of the blanks and, if so, which parameters guided them; 2) the technical responses to the different qualities/limits of the raw materials; 3) if technical variability reveals occasional choices or an invariant knowledge independent from the several raw material constraints^18^. Four morphological types has been defined in frontal view (ovoid, sub-quadrangular, subtriangular, and subcircular) and four in lateral view (flat, bi-convex, plano-convex, and concavo-convex). We recorded length, width, thickness, and weight of each specimen. According to the Wentworth grain scale classification^83^, pebbles are comprised between 4 and 63 mm and cobbles between 64 and 256 mm.

We classified cores according to 1) technique; 2) the number of flaking surfaces; 3) the direction of flaking; 4) the presence/absence of a distinct striking platform; 5) the features of the striking platform; 6) the angle between the striking platform and the flaking surface; and 7) the angle(s) between/among flaking surfaces. Considering these features, core analysis allows us to identify exploitation modalities and volume management^10,11,15,18,24^.

The flake analysis takes into account the dimensions (length, width, and thickness measured according to the flaking axis), the number and direction of negative scars on the dorsal face, the type of butt, the shape and cross-section, the correspondence between morphological and flaking axis, the presence of overshot/hinged removals, the presence of retouch, the location and type of retouch, and possible correspondence among shapes, sizes and flaking methods^10,11,15,18,24^. As Hayden^53^ has noted, the identification of bipolar-on-anvil flakes relies on the presence of several diagnostic patterns, not all of which need to be present. It is therefore not possible to unquestionably distinguish bipolar-on-anvil from freehand flakes, especially in the case of *entame* (opening) flakes and of cortical flakes with flaked butts. However, among flakes with unidirectional removals on the dorsal face we have isolated some distinctive technical traits, sufficient to separate them from the other flakes with the same negative scar pattern belonging to freehand percussion. They are listed in the section devoted to flake description.

About bladelet-like flakes, Bordes^84^ refers to blade/bladelet when the length of a flake is at least equal to twice its width and adds (p. 6) that “English-speaking authors, among others, make a distinction between true blades and blade-like flakes, a true blade showing traces of previous parallel removals on its upper face, and also having more or less parallel edges. Although the distinction is perfectly valid in theory, it is often difficult to make in practice, and will therefore be disregarded”. Tixier^85^ introduces for bladelets of the Epipaleolithic of Maghreb the limit of length equal to 50 mm and limit of width equal to 12 mm. In this work we refer to Bordes’ definition and we consider these products bladelet-like flakes because length/width ratio is not exactly 2, but it is only approaching 2.

## Supplementary information

Supplemental information.
