# Supplementary material for: Dedicated core-on-anvil production of bladelet-like flakes in the Acheulean at Thomas Quarry I - L1 (Casablanca, Morocco)
Source: Sci Rep. 2020 Jun 8;10:9225. doi: 10.1038/s41598-020-65903-3 (PMC7280310; doi:10.1038/s41598-020-65903-3)
Supplement: Supplementary file 1 — Supplemental information. [file 41598_2020_65903_MOESM1_ESM.pdf]

Supplementary Information

Dedicated core-on-anvil production of bladelet-like flakes in the Acheulean at Thomas Quarry I - L1 (Casablanca, Morocco)

Rosalia Gallotti[1,2,*], Abderrahim Mohib[3], Paul Fernandes[2,4], Mohssine El Graoui[5], David Lefèvre[1], Jean-Paul Raynal[2,6]

[1]Université Paul Valéry Montpellier 3, CNRS, LabEx Archimede - ANR-11-LABX-0032-01 - and UMR 5140 Archéologie des sociétés méditerranéennes, Campus Saint Charles, 34199 Montpellier (France)

[2]Université Bordeaux 1 UMR 5199 PACEA-PPP, Bâtiment B18 allée Geoffroy Saint-Hilaire CS 50023 F - 33615 Pessac Cedex (France)

[3]Centre d'interprétation du Patrimoine du Gharb, Direction provinciale de la Culture, Quartier administratif, Bd Mohamed V, Kenitra (Morocco)

[4]SARL Paleotime, 6173 rue Jean Séraphin Achard Picard, 38250 Villard-de-Lans (France)

[5]Institut National des Sciences de l'Archéologie et du Patrimoine, Madinat Al Irfane, Angle rue N°5 et rue N°7, Rabat-Institut, BP 6828, Rabat (Morocco)

[6]Department of Human Evolution, Max Planck Institute for Evolutionary Anthropology, Deutscher Platz 6, 04103 Leipzig(Germany)

*rosaliagallotti@yahoo.it

1. Litho-chrono-stratigraphy

In the New Casablanca Stratigrafic Scale, four formations, including several members, ranging from the late Early Pleistocene to the Late Pleistocene have been defined: the Oulad Hamida Formation (late Early Pleistocene - early Middle Pleistocene), the Anfa Formation and the Kef Haroun Formation (Middle Pleistocene), and the Dar Bou Azza Formation (Late Pleistocene) (Fig. S1). Their age estimates have been established by lithostratigraphy, biostratigraphy, absolute dating (OSL, ESR, U/Th), aminochronology and are currently systematically investigated for paleomagnetism after preliminary attempts[1-6].

Thomas Quarry belongs to the Oulad Hamida Formation[7-13]. The Oulad Hamida Formation has been first described in Thomas Quarry I and then in Oulad Hamida 1 Quarry[14-16]. The Oulad Hamida Formation (OHF) is based on a major unconformity formed at an altitude of around 28 m asl (general leveling of Morocco) at the expense of the Cretaceous or Palaeozoic substratum.

The OHF includes five members, OH1 to OH5, from base to top[15]. A member is defined by a unit sequence, characterized by a succession of intertidal, supratidal, and dunal or continental deposits bounded at its base and top by unconformities or erosional surface. It records highstand sea levels associated with the formation of a shoreline marked by a cliff. The unconformities or sequence boundaries that separate them sign the regression of the ocean and the continentalisation of the coast. The OHF Members are thus correlative of cyclic sea level fluctuations in relation to global glacio-eustatic changes and the extent of glaciations at high latitudes, preserved thanks to regional tectonic uplift[2,14].

Considering than the Oulad Hamida Formation predates the Anfa Formation, which members correlate to MIS 11, 13 and 15, thus, the OHF records at least four highstand sea-levels, probably MIS 17 to 25 if the record is complete, more if it is not, pushing it back from the late Early Pleistocene to the very beginning of the Middle Pleistocene[2,6,14,15].

Their lithostratigraphy is as follows, from base to top:

***OH1 Member**:* Bed 1 is composed of a coarse calcirudite at the base and a coarse, stratified biocalcarenite; intertidal depositional environments.

Bed 2 – Unit L is a 2 to 3 m succession of yellow lenticular limestone beds with cross-bedding structures, composed of microsequences of mudstones, intraclast sands separated by emergent surfaces formed in a continental fluviolacustrine hydrosystem with shifting channels and a temporary water table, followed by pedogenised aeolian sands. First tests using OSL dating provide an age of between 0.8 and 1.2 Ma for the unit L limestone deposits[4] which should thus belong to the Matuyama chronozone, an attribution still to confirm. Thus, an age of c. 1 Ma is for now the best estimate for Bed 2, either before or immediately after the Jaramillo subchronozone.

OH2 Member: Overlying an erosion surface above the OH1 Member deposits, there are coarse biocalcarenites with curved cross-bedding followed by finer inclined planar-bedding biocalcarenites formed within intertidal depositional environments; vertically there follows massive banks of aeolianites about ten metres thick, their upper part affected by fersialsol pedogenesis. The OH2 intertidal deposits register a highstand sea level followed by the regression of the ocean, which is conveyed by the formation of aeolianite.

OH3 Member: This is composed of coarse and/or coquinoid biocalcarenites (with inclined planar bedding), overlying an abrasion platform with associated calcarenite and lacustrine limestone blocks and pebbles that truncate the deposits of OH1 and OH2 Members and cut a cliff into the OH2 Member deposits, the base of which sits at an altitude of 37 m above sea level (asl). The OH3 deposits register a highstand sea level associated with the formation of a palaeoshoreline. The regression phase that follows is coupled with the formation of aeolianite.

Several formations - OH4 and OH5 Members - are associated with a complex of palaeoshorelines cut into the earlier OH1, OH2 and OH3 Members;

OH4 Member: Composed of fine, grey, planar bedded calcarenites with, at the bottom inside the cavities, calcirudite mixed with blocks from earlier formations; these intertidal facies, which can be seen at an altitude of up to 34 m asl, are associated with the formation of the palaeoshoreline with deep cavities.

As continental fillings of these cavities have been dated to 0.5 to 0.7 Ma[4,10-12], OH Members 4, 3, 2 and 1 representing previous highstand sea-levels are thus undoubtedly much older.

OH5 Member: it is an aeolianite made up of layers of weakly cemented grey sands, with a pronounced cross-bedding. These deposits are associated with a palaeoshoreline cliff, which partly follows the same path as the one associated with OH4 Member.

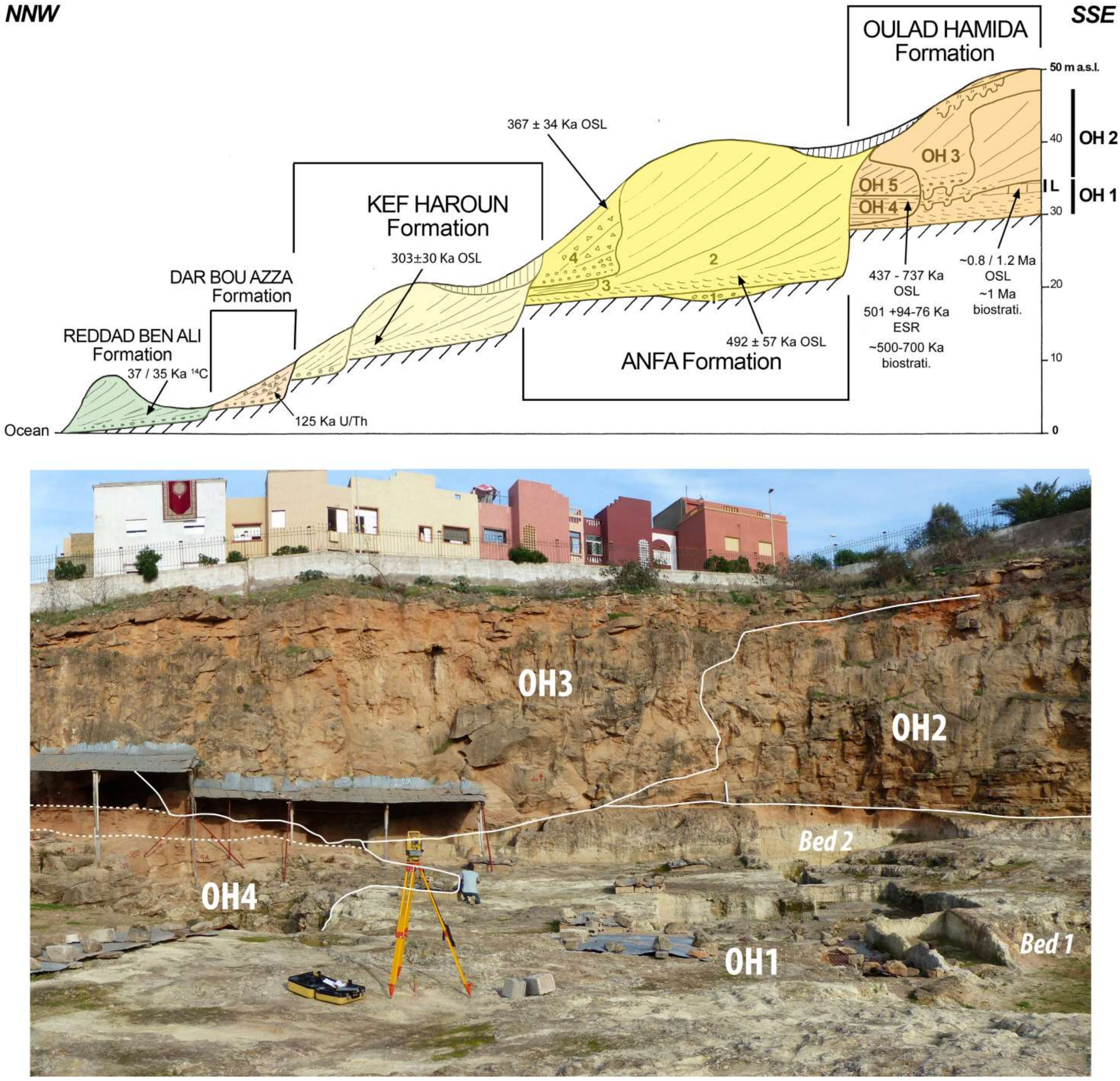

Figure S1. Top: synthetic stratigraphic section of the Quaternary formations of the SW area of Casablanca (drawing by D. Lefèvre). Bottom: Member succession in the Oulad Hamida Formation at Thomas Quarry I (photo and drawing by D. Lefèvre).

References

1. Lefèvre, D., Texier, J.-P., Raynal, J.-P., Occhietti, S. & Evin, J. Enregistrements-réponses des variations climatiques du Pléistocène supérieur et de l'Holocène sur le littoral de Casablanca (Maroc). *Quaternaire* **5,** 173-180 (1994).

2. Lefèvre, D. Du continent à l'océan. Morphostratigraphie et paléogéographie du Quaternaire du Maroc atlantique. Le modèle casablancais. Habilitation à diriger des recherches, vol. 3, Université de Montpellier III (2000).

3. Occhietti, S., Raynal, J.-P., Pichet, P. & Lefèvre, D. Aminostratigraphie des formations littorales pléistocènes et holocènes de la région de Casablanca, Maroc. *Quaternaire* **13**, 55-64 (2002).

4. Rhodes, E.J., Singarayer, J., Raynal, J.-P., Westaway, K. & Sbihi-Alaoui, F.-Z. New age estimations for the Palaeolithic assemblages and Pleistocene succession of Casablanca, Morocco. *Quat. Sci. Rev.* **25,** 2569-2585 (2006).

5. Texier, J.-P., Lefèvre, D. & Raynal, J.-P. Contribution pour un nouveau cadre stratigraphique des formations littorales quaternaires de la région de Casablanca. *C. R. Acad. Sci. Paris, série II,* **318**, 1247-1253 (1994).

6. Texier, J.-P., Lefèvre, D., Raynal, J.-P. & El Graoui, M. Lithostratigraphy of the littoral deposits of the last one million years in the Casablanca region (Morocco). *Quaternaire* **13**, 23-41 (2002).

7. Raynal, J.-P., Magoga, L., Sbihi-Alaoui, F.Z. & Geraads, D. The Earliest Occupation of Atlantic Morocco: The Casablanca Evidence in *The earliest occupation of Europe* (eds. Roebroeks, W. & van Kolfschoten, T.) 255-262 (University of Leiden, 1995).

8. Raynal, J.-P. *et al.*The earliest occupation of North-Africa: the Moroccan perspective. *Quat. Int.* **75,** 65-75 (2001).

9. Raynal, J.-P. *et al.* Casablanca and the earliest occupation of North Atlantic Morocco. *Quaternaire* **13***,* 65-77 (2002).

10. Raynal, J.-P. *et al.* Hominid Cave at Thomas Quarry I (Casablanca, Morocco): recent findings and their context. *Quat. Int.* **223-224,** 369-382 (2010).

11. Raynal, J.-P. *et al.* Contextes et âge des nouveaux restes dentaires humains du Pléistocène moyen de la carrière Thomas I à Casablanca (Maroc). *Bull. Soc. préhist. fr.* **108**, 645-669 (2011).

12. Raynal J.P., Lefèvre D., El Graoui M., Geraads, D. & Rué, M. La Grotte des Rhinocéros (Casablanca, Maroc): le remplissage et son âge in *Préhistoire de Casablanca. I - La grotte des Rhinocéros (fouilles 1991 et 1996)* (eds. Raynal, J.-P. & Mohib, A.) 79-86 (Institut National des Sciences de l'Archéologie et du Patrimoine, VESAM volume VI, 2016).

13. Raynal, J.-P., Gallotti, R., Mohib, A., Fernandes, P. & Lefèvre, D. The western quest, First and Second Regional Acheuleans at Thomas-Oulad Hamida Quarries (Casablanca, Morocco) in *Vocation préhistoire. Hommage à Jean-Marie Le Tensorer* (eds. Wojtczak, M. *et al.*) 309-322 (ERAUL 148, 2017).

14. Lefèvre, D. & Raynal, J.-P. Les formations plio-pléistocènes de Casablanca et la chronostratigraphie du Quaternaire marin du Maroc revisitées. *Quaternaire* **13***,* 9-21 (2002).

15. Lefèvre, D., Raynal, J.-R. & El Graoui, M., Le Quaternaire d'Oulad Hamida in *Préhistoire de Casablanca. I - La grotte des Rhinocéros (fouilles 1991 et 1996)* (eds. Raynal, J.-P. & Mohib, A.) 45-59 (Institut National des Sciences de l'Archéologie et du Patrimoine, VESAM volume VI, 2016).

16. Lefèvre, D. & El Graoui, M., Les formations pléistocènes de la carrière Oulad Hamida 1 in *Préhistoire de Casablanca. I - La grotte des Rhinocéros (fouilles 1991 et 1996)* (eds. Raynal, J.-P. & Mohib, A.) 61-77 (Institut National des Sciences de l'Archéologie et du Patrimoine, VESAM volume VI, 2016).

2. Mineral resources used by humans at Thomas Quarry I - L1

Ancient excavations at Casablanca have revealed that quartzites were the main raw materials exploited by prehistoric humans along with few flints[1]. This is also true at Thomas Quarry I-L1 (ThI-L1) where the used materials can be divided into two main families: the tough rocks from the local basement, including mainly quartzites, and flints, varied in appearance but very poorly represented. The Casablanca sequence is known to have Pleistocene marine deposits rich in small flint pebbles, such as member 4 of the Anfa Formation (former "Anfatien" G2 of Biberson, Middle Pleistocene, MIS 11)[2] and the Dar Bou Azza Formation (Late Pleistocene, MIS 5). Moreover, there may have been many others secondary deposits easy to collect by humans; this scarce resource available in small modules (less than 10 cm) was used at different times in the regional Acheulean sequence in a low proportion. In the very close mineral environment of the site, a few hundred meters or even less, suitable rocks were therefore available in abundance for flaking.

2.1. Quartzites

The term "quartzites" includes several varieties of Cambrian-Ordovician basement rocks that form the bulk of the Casablanca anticlinorium and are essentially Acadian and Arenig in age[3]. At the base of the Acadian, there are shales, siltstones, micaceous and chlorite sandstones (Bouznika shales), then arkozic feldspathic arenites and feldspathic grauwackes interspersed with fine sandstones that form a bar in the landscape (El Hank “quarzite” formation)[4-6]; on the roof of the Acadian, more or less coarse green schists, grauwackes and arenites form the Dar Bou Azza series[7,8]. These Cambrian deposits are interspersed with volcanic facies, flows, tuffs and conglomerates with volcanic pebbles[9]. The Ordovician is represented by a thick series of more than 1000 m, cut by Wadi Nefifikh: conglomerates, chlorito-thinned pelites, fine sandstones or quartzites form bars[9-11].

These "quartzites" are low-porous, dense rocks with variable mechanical properties depending on bedding, cracking and alteration. They are the substrate of the Mio-Plio-Pleistocene formations of the Casablanca sequence. They were exposed on the foreshores or were locally preserved in points; notched by the wadis and Quaternary paleo-rivers, they largely supplied the alluvium of the wadis and the various ancient beaches with pebbles/cobbles of various modules. Even today, the raw material is still available in the form of whole or naturally fractured cobbles: from Casablanca to Dar Bou Azza (Fig. S2), there is a diverse range of supports on today's beaches (whole pebbles/cobbles, broken pebbles/cobbles, pebbles/cobbles fragments, natural blocks and chips).

We have distinguished a light grey facies, very well crystallised, with conchoidal fracture and mechanical properties very close to those of flint and largely outcropping between El Hank and Sidi Abderrahmane, and some less silicified and micaceous varieties, less resistant to weathering, greenish to purple and available in the form of cobbles. Some other rocks in very small quantities evoke eruptive facies, trachytes, dolerites, basalt, cinerites and volcanic tuffs, which outcrop in the vicinity of Casablanca in the Cambrian, Ordovician and Permo-Triassic periods[12,13] and have been remobilized in river alluvium and marine beaches.

2.2. Flint

The method which has been developed and applied to characterize flint origin, transport mechanisms, and arrival in the site (by natural agents or by humans) is the only one and pioneering method which is currently giving robust informations[14-19]. Informations decoded on flint surfaces and cortex explain pre and post-sedimentary processes which affected flint nodules then artefacts, allows to understand whether flint arrived naturally in the site or was gathered elsewhere by humans and precisely where and finally give clues about the site taphonomy. We studied 95 artefacts from ThI-L1 after a detailed geological survey and the observation of several geological samples.

Flint did not format Casablanca and primary deposits only outcrop in the hinterland[20,21] and have fed the secondary deposits located in the drainage system towards the Atlantic coast. To the south and south-east of Casablanca, flints are present from Chichaoua region to the Khourigba area, in the Upper Cretaceous

(Maestrichtian) and Eocene (Thanetian, Ypresian, Lutetian)[22-24]. In the Tadla, the Upper Cretaceous (Turonian) is represented by limestone with flint and between Melgou and Oued Zem, three flint lines have been reported in the Senonian[25]. In the Atlas, the Upper Cretaceous (Turonian) is also represented by limestones with flint. In the East, in the Middle Atlas, Feleddi marls with flint are dated to the Maestrichtien-Danien; we also know the dolomitic limestones with flint at Djebel Hayane and to the south of the latter, the silicified limestones and marls with flint in the Aouam plain[25]; the limestones with flint of the Midelt region have probably only supplied secondary sites of the drainage network towards the Mediterranean. In the northeast of Casablanca, in the Rabat region, the Lower Devonian contains primary flint nodules, the wadis provide secondary alluvial deposits and the ancient alluvial deposits of the Rabat plateau ("Moghrebian" of the former authors) also provides abundant flint pebbles[26-30]. Further on, the pre-Rif formations offer primary localities in the white marls of the Bartonian-Lutetian[14].

A revision of all the sources available in the Casablanca hinterland revealed two types of flints: the MF15 type, present on the phosphates plateau from the top of the Maestrichtian to the roof of the Montien and the MF25 type, frequent from the Ypresian to the base of the Lutetian. We had previously identified these flints, which are more or less rich in phosphates, in Wadi Erguita regions east of Agadir, in the Meskala region east of Essaouira, in the Oulad-bou-Sbaa area around Chichaoua and finally in the Ganntour region south of Youssoufia (Fig. S2). We will confine ourselves here to the study of phosphate flint between Khourigba and Casablanca, which accounts for all the facies observed in a dynamic perspective[14]. The characterization method used is based on the fact that the mineralogy and morphology of a flint reflect its genesis and post-genesis residence conditions. The creation of a geo-referenced geological database several years ago and adapted to the taphonomy of local siliceous rocks, allows us not only to specify the collection sites used by prehistoric peoples, but also to determine the primary deposits at the origin of the highly transformed silicifications found in an archaeological context. We recognized in ThI-L1 layer four types of flint: MF4, MF15a, MF15b and MF25.

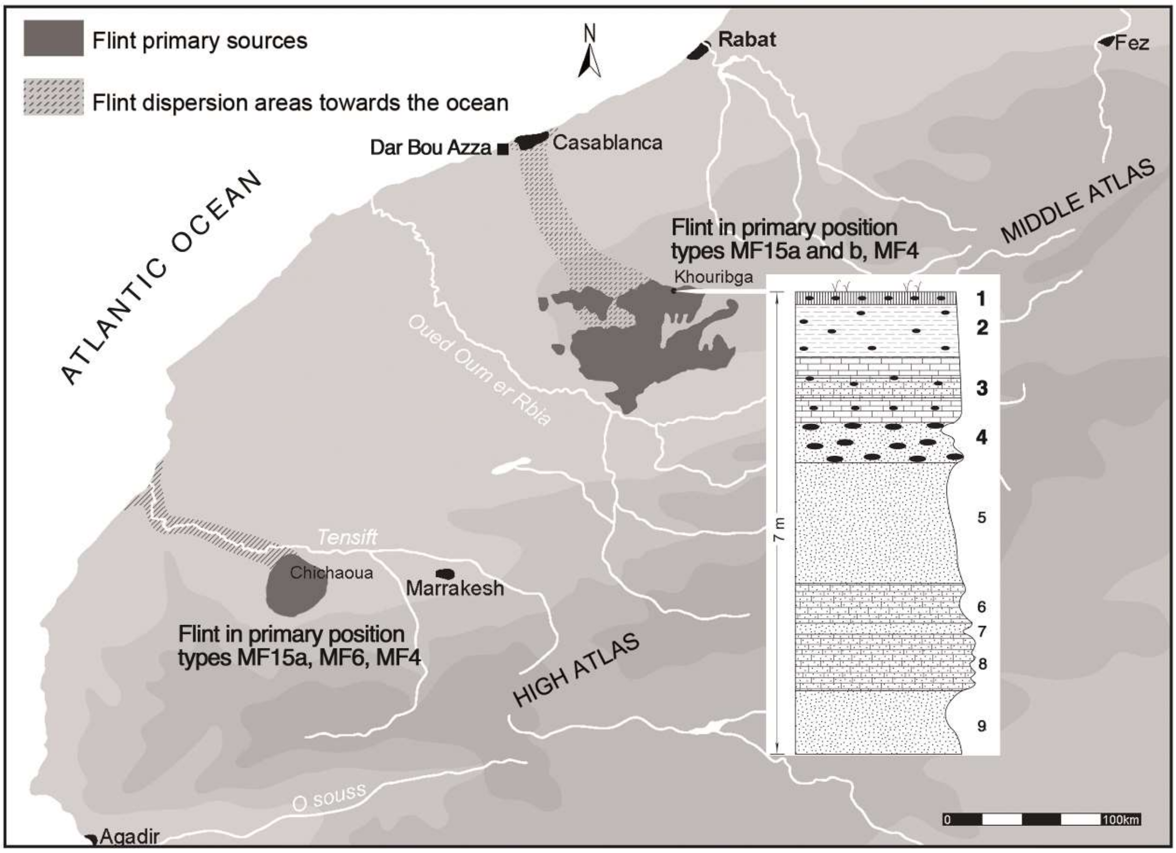

Figure S2. Primary sources and dispersion areas of phosphated flints with log of formations at Mrizig near Khouribga (1 to 9, see details in the text). Drawing by P. Fernandes and J.-P. Raynal. This map was generated on the basis of scanned paper archives of the authors with Adobe Photoshop CC version 2015-1, 20151114.r301 X64. Copyright 1990-2015 Adobe Systems Incorporated and its licensors. All rights reserved.

2.2.1 Theory and methods

The genesis of all these flints is a siliceous cementing of phosphate sands more or less rich in carbonates. Their study shows a preponderance of microcrystalline calcedonite over quartz. This factor will evolve during the post-genetic phase, especially in the figurative elements. The study of the mineral evolution of flint, from its source to the coast, makes it possible to distinguish evolutionary stages until its complete transformation. We therefore followed several flints of Moroccan phosphates, from primary deposits (the northeastern part of the Oulad-Abdoun, Khouribga) to Mesetian marine sediments around Casablanca.

We used two protocols: one for primary deposits and the other for all types of secondary deposits. For the primary deposits, we carried out a detailed sampling of the outcrops, respecting the position and polarity of the silicification types. For secondary deposits, the protocol is based on a statistically significant collection (50 samples per deposit). For each of them, we have defined the mode of transport and deposition that are at the origin of the surface formation that contains them. We collected surface samples, in flint silts and underlying Lutetian and Ypresian limestones[22] west of Khouribga between Bled Sahel and Mrizig. The second collection area is located in the alluvium of one of the tributaries of the Oued Mellah river that flows into the ocean just north of Casablanca. Finally, the last sampling area is located on the coast at Dar Bou Azza (15 km south-west of Casablanca), on the present day beach and in the conglomeratic Dar Bou Azza Formation deposited during MIS5.

In the laboratory, thin sections (30 µm thick) cut from phosphate flints were analysed to decipher the evolutions along the Khouribga/Casablanca route (in the primary deposit, in the hydrographic network and on the coast). For the determination of bioclasts, we made thin sections 300 µm thick. In parallel, a study at the MEB (Jeol JSM-6460 LV) was carried out using secondary electrons under 20KV voltage after gold plating on the surfaces and internal zone of flint collected along the route: in the primary deposits west of Khouribga, in the alluvium of the tributaries of the Oued Mellah and finally on the Meseta coast. The magnifications were between x 350 and x 20 000. Ultra-microscopic analyses of the internal areas were carried out on fresh fracture surfaces. This series of observations is based on a preliminary analysis of all samples with a binocular magnifying glass. Twelve thin sections were performed on selected samples and studied under an optical microscope. Phases interpretation required the use of the EDX (Energy Dispersive X- Ray Analysis) microprobe. This work was carried out with the JEOL scanning electron microscope of CRP2A Bordeaux 3 University, equipped with an INCAx-sight energy dispersion spectrometer (Oxford industries).

2.2.2 From Khourigba to Casablanca: a dynamical evolution

In the Khourigba basin, subsidence began to appear early in the Upper Cretaceous and ended in the Upper Lutetian. Phosphates genesis and carbonates sedimentation reached here their greatest development. The layers the richest in flint (MF15a) are present around Khouribga in a pinkish sandy limestone rich in phosphates dated to mid-Lutetian. This type consists of large irregular nodules with a fluidal structure and with phosphates pseudooliths dispersed in the matrix as far as the core (Fig. S3: 1). Phosphates developed because of syn-sedimentary changes[24]. It is worth mentioning the presence of a large number of the same flint in secondary positions (types MF15a2 and MF15a4) in the Miocene and Quaternary formations at the top and on the western edge of the phosphates plateau. These different sources represent the main source that fed part of the Mesetian coast through Wadi Mellah and its tributaries, at least since the Miocene.

2.2.2.1 The flint in primary position

The Khouribga area was the first mined for phosphates in Morocco but its reserves are now exhausted. We collected flints in primary and secondary positions on the plateau between Khouribga and Mrizig (Fig. S2) which forms the interfluve between Wadi OumEr-Rbia and WadiMellah basins. Several units were defined after Maastrichtian globigerines. The Maastrichtian outcrops on the edge of the plateau (Fig. S2: 9) and is overlain by white marl limestones dated to the Ypresian (Fig. S2: 6,8)[24]. Above, grey sandy phosphates deposited. The Ypresian is identified by coarse fossiliferous sandy layers (Fig. S2: 5) and by its important phosphate complex (Fig. S2: 4) which contains flints (MF15b) and which would indicate the summit of the

Ypresian and the base of the Lutetian[24]. Lutetian in this sector is represented by pink sandy limestones with phosphate pockets and brown flint nodules of type MF15a (Fig. S2: 3). In the upper part of this unit, a shelly limestone most often silicified occurs in some places. Associated with this type of silicification, we note the presence of decimetric to pluri-decimetricgeodes of calcedonite (MF4 type); they are numerous in the Lutetian in the northern part of the plateau. The series continues above with red clays (sometimes phosphatic) with flint resulting from alteration (Fig. S2: 2) during which carbonates were dissolved and replaced by clays. The flint present in this unit is a slightly evolved form of Lutetian's MF15a. At the top of the series, the modern soil also contains flint with more pronounced alteration stigmata (Fig. S2: 1).

The majority of these flints are characterized by their high granular phosphates content. Their structure has an homogeneous to zoned appearance. They are dark brown to grey with a packstone texture, rich in phosphates pseudooliths, elongated bone debris and coproliths. These pseudooliths are opaque and most often grey or pigmented in red brown. The elements float in the matrix and there is no preferential orientation. The microscopic structure of phosphates varies: homogeneous grains, grains with concentric structures with or without nucleus and finally, cleaved automorphic crystals. In some grains, calcedonite and microquartz have replaced phosphates (Fig. S4: 1). Bone debris are often silicified. Microquartz between 5 and 10 µm in s represents the latest filling phase. We have noted the presence of flabelliform spherolites of chalcedonite and some rhombohedral boxworks (lined with microquartz). These indicate the presence of carbonates in the original composition. Finally, megaquartz is rare and fill large bioclasts such as bivalves. The matrix is dark and translucent to matt. It consists mainly of microcrystalline/cryptocrystalline calcedonite in patches where the figurative elements are more dispersed.

2.2.2.2 Flints in the hydrographic network

We first collected flint from the recent alluvium of Wadi Zamrina, one of Wadi Mellah tributaries which runs along the northern edge of the plateau and found types MF15a, MF15b and MF4 (Fig. S3: 2). Their neo-cortex is siliceous. The surfaces are irregular, controlled by differential dissolution. Phosphates and bivalve tests appear in relief. There is a silica film in some places which gives to the surface a smooth and glossy appearance. The original brown colour is bleached by loss of material by dissolution. This process of alteration obliterates the older torrential stigmata. Some samples (MF15a) have cups and corrosion ranges. The matrix is identical to those of the samples taken upstream in primary deposit. At this stage of the itinerary, only phosphate grains are sensitive to alteration and most of them near the periphery of the block have disappeared, leaving gaps. At this stage only the surface of flint has evolved. There is a minimal transformation of the mineralogical composition of the internal zone with only the disappearance of phosphate grains in the subcortical zone.

2.2.2.3 Flint in coastal formations

Two formations rich in flint have caught our attention: a fossil littoral deposit at Sidi Abderrahmane Quarry ("Anfatian") and the more recent deposits at Dar Bou Azza (MIS 5 to present beach). They contain very evolved forms of Ypresian and Lutetian flints of the phosphates plateau (Oulad Abdoun, Khourigba area), located about 100 km to the east.
On the basis of the degree of alteration of the flint pebbles from the marine formations, two major families can be distinguished: one is very highly transformed and the other is lesser evolved. All flints examined show altered surfaces and these transformations were controlled by sedimentary and climatic processes along the flint route. The late evolution is well developed and controlled by the marine environment. The associations of previous stigmata acquired in the hydrographic network are most often obliterated by the heavy transformations imposed by the marine environments: first in infratidal (high energy) and then in intertidal (more static) conditions. Some similar observations were made on the quartz grains in Temara caves[31], a hundred kilometres north of Casablanca.

Flint in coastal formations: their subcortical zone
The superficial characteristics and subcortical transformation phases of geological samples and archaeological objects studied make possible to distinguish types of neo-cortex.

- Category 1: the surface is irregular with a deep white patina. It bears the traces of an ancient aquatic phase that has not been too much transformed. There is no evidence of recent V shocks and dissolution. The virtual absence of coloured patina makes could indicate an origin close to the seashore.

- Category 2: the surface is irregular, with stigmata from an old aquatic phase with a strong polishing gradient which is notched by a series of violent shocks, themselves polished. Rare dissolution patches are noted. The yellow patina is probably related to the oldest aquatic phase. The ancient aquatic stigmata are clearly visible, but the alteration gradient is strong. The yellow patina still present allows to attribute this old series of shocks (V-shaped shock, cups) to a fluvial phase. This phase - or these old phases - are notched by medium-size V-shaped shocks with a slight blunt gradient. These stigmata of marine origin are associated with phases of dissolution of marine origin too. This type of surface is associated with matrix that are very poorly recrystallized. Sometimes the bleaching is complete and obliterates the initial texture. Some of the matrix retain their original colors.

- Category 3: the surface is smooth and clean, usually with a thick uniform white patina that homogenizes the periphery of the object. A very discreet ancient water transport phase can be inferred, notched by rare traces of recent shocks. This type is common in the indurated conglomerate found at Dar Bou Azza. It is therefore a fossil facies. The surface is opaque. Recent V-shaped shocks are rare. Only a few very old V-shaped shocks are present in the most exposed areas. We observe the presence of shocks on the late exposed face, associated with dissolution marks, while the very old traces of V-shaped shocks are preserved on the other face. No dissolution was observed. This cortical type is totally newly formed and thicker than the previous types. There is nothing left of the original peripheral texture. Depressions are very rare and sometimes retain traces of the ancient transport or immobilization stages.

- Category 4: the surface is more or less regular. The general aspect remains close to category 3. The presence of isolated patches of silica deposits that cover older surfaces justifies the distinction. In addition we note the absence of dissolution.

- Category 5: the surface is smooth but has many dissolution ranges. As in category 3, the old aquatic surface has completely disappeared. The sample has acquired an almost homogeneous morphology. A strong alteration gradually transforms the surface which appears poorly cleaned, the perfect previous smoothing gives place to a new irregular surface.

- Category 6: this is the most advanced alteration phase. The siliceous film has disappeared, the surface is lumpy to saccharoid. This is a necrotic facies: you can scratch the surface with the nail. The matrix has lost almost all of its density. Necrosis is important and the texture is not identifiable. This is the only facies to which we cannot give an exact origin.

<u>Flint in coastal formations: their internal zone</u>

The internal zone is of centimetric size and has a texture different from that of the primary deposits flints. The texture is frequently mudstone. The matrix remains mainly calcedonic. However, some phosphated flints (the most evolved of the MF4 or MF15a types) are essentially made of quartz.

Two types of matrices are observed, indicating distinct replacement processes depending on the initial textural framework of the altered materials. The original large spherolites evolve towards petaloid megaquartzes (Fig. S4: 3). On the other hand, the disordered micro-calcedonite patches seem to evolve towards a more or less mosaic texture with well-formed quartz microcrystals. These types of transformation involve a process of recrystallization. These replacements, or reorganizations, partially preserve the previous microstructures. Thus, at the expense of areas that lose their soluble matter, fibrous forms of silica first develop followed by crystallised forms. There are two categories:

- Category a: the quartz/calcedonite ratio did not change;

- Category b: the quartz/calcedonite ratio is greater than 1. The megaquartz crystal patches are part of the composition. This type of texture was totally absent in the facies sampled in the primary and sub-primary

zones. Calcedonite pherolites are in minority and the ancient mineral forms (phosphates and flurapatite) are mostly pseudomorphosed into silica. This facies is close to the flint textures in the hydrographic network but differs by its reduced amount of calcedonite, attesting of its vulnerability in a marine environment.
The mineralogical evolution of MF15a is marked by an increase in microquartz and megaquartz in-filling figured elements and cracks. Traces of sedimentary bedding, phosphate grains, cleaved phosphate, calcedonite pherolites and geodic quartz are characteristic of the phosphated flints of the Khouribga plateau. The texture becomes more and more homogeneous. These facies are very often associated with a totally or partially reorganized matrix. Moreover, most of them have lost their initial color and have become totally colourless. The traces of oxidation are concentrated on the face bearing the shocks and striations and more discreetly throughout the subcortical area.
There are many boxworks, these volumes are lined bymicroquartz crystals that surround macroquartz in the center (Fig. S4: 4). This is another discriminating feature to recognize flint which travelled in marine environment because the boxworks of flint collected in colluviums remain completely empty. As for flints collected from alluvial deposits, the systematic presence of megaquartz is a relevant marker to distinguish flints which travelled from those collected in sub-primary or colluvial positions.

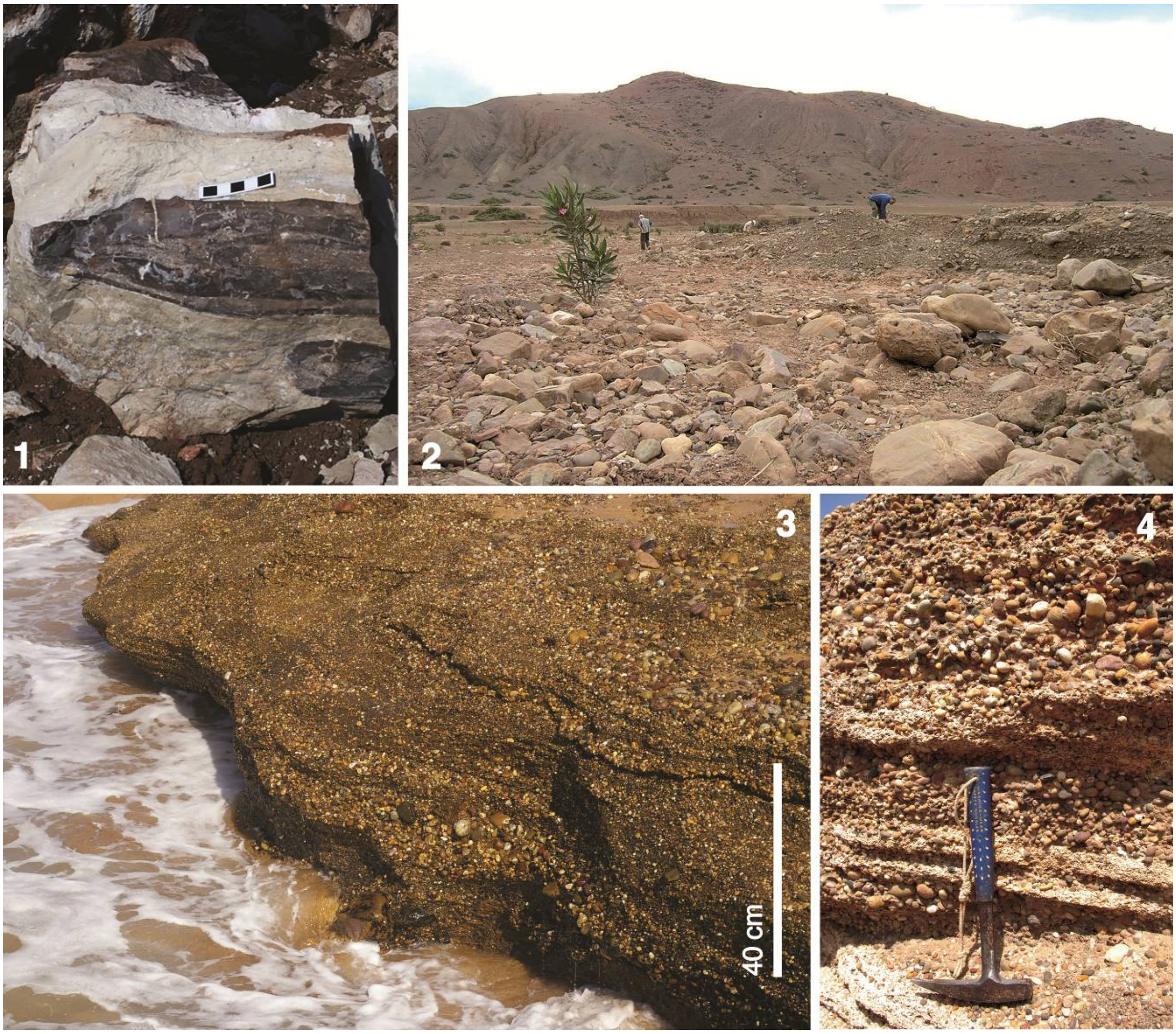

Figure S3. Type MF15a nodule in the Lutetian at Khouribga (photo by P. Fernandes). 2: alluviums with flint in Wadi Zamrina, east of Casablanca (photo by J.-P. Raynal). 3: MIS5 deposits with flint pebbles at Dar Bou Azza (photo by D. Lefèvre). 4: Gravels with flint pebbles in the Anfa Formation member 3 (“Anfatian”) at Sidi Abderrahmane Quarry (photo by D. Lefèvre).

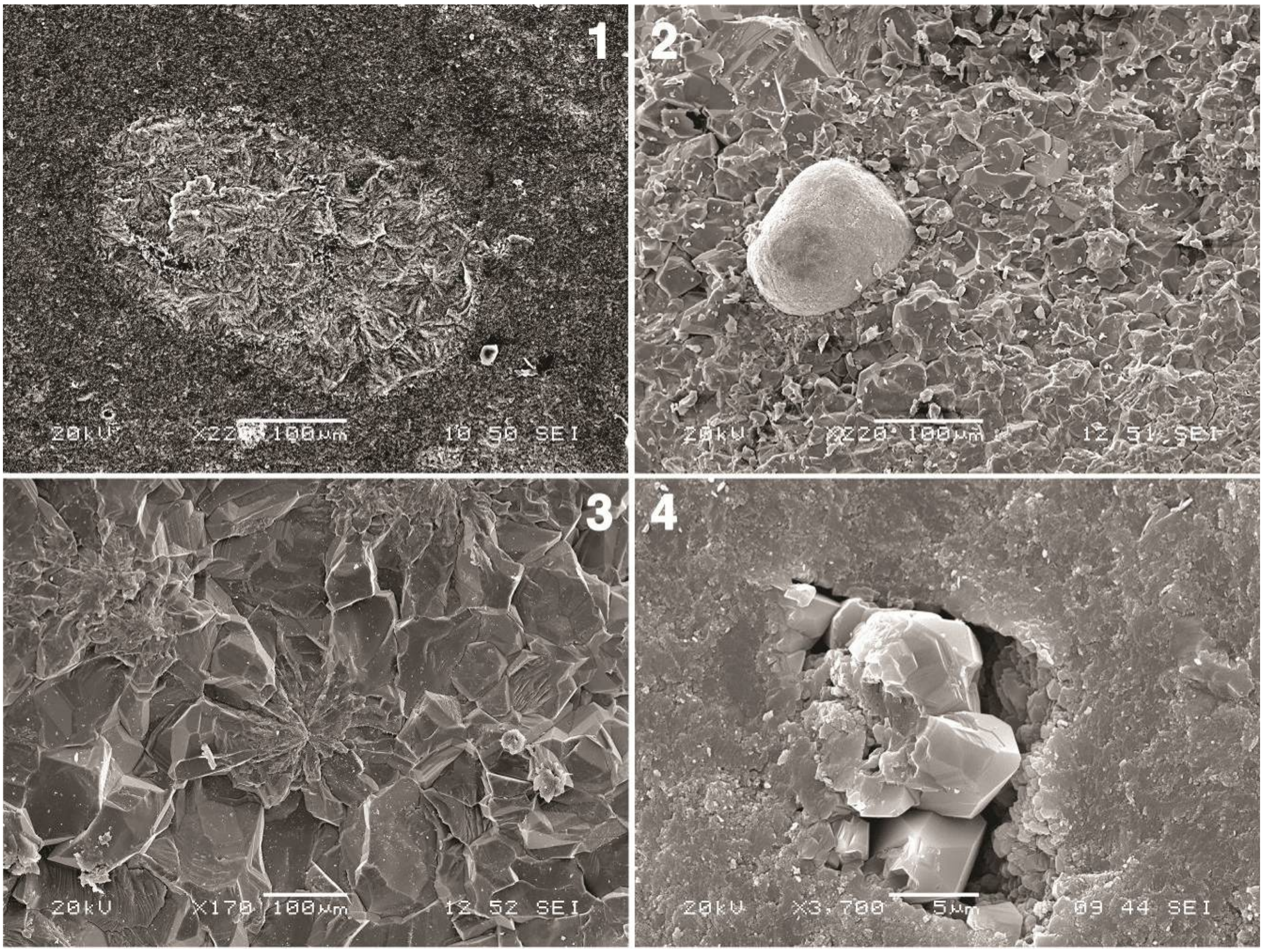

Figure S4. Scanning electron microscope images. 1: type MF15a, phosphate grain replaced by calcedonite and microquartz. 2: MF15a, Dar Bouazza beach deposits, preserved phosphate grain. 3: MF15a, Dar Bouazza beach deposits, large spheroid evolving towards quartz. 4: MF15a, boxwork with quartz infilling (photos by P. Fernandes and F.X. Le Bourdonnec).

2.3 Conclusion

The various quartzites and other tenacious rocks form the bulk of the lithic raw material supplies in TH1-L1 and there is no doubt that they were preferentially collected locally.

Flints collected show significant transformations in continental environments and especially in aquatic environments. All were transported over the same distances by different vectors and regimes and were integrated into the marine environment at different times. These chemical and chronological mechanical factors are at the origin of the different textures encountered. On the surfaces of the flint studied, the alteration processes regularly outweigh the mechanical characteristics of the aquatic type. It seems that a resumption of mechanical processes generating V-shaped shocks is interspersed between an old active phase, followed by the formation of a film representing the terminal phase of evolution. The flint comes from secondary deposits of the same type where small pebbles (from 40 to 70 mm maximum) meet, with similar flattening, blunt and asymmetry indexes. Almost all of it was collected in the coastal zone, the site of a low intensity mixing (supratidal and/or infratidal). The microreliefs preserved on the natural face of the artefacts are comparable in every respect to the pebble surfaces we collected on the present-day beaches not far from the site.

In conclusion, the four types of flint found at ThI-L1 reveal a long stage in marine environment, which has been heavily erased by posterior alteration. This means 1) that the pebbles derive from marine deposits very

close to their final deposition place (THI-L1 deposit) and that a successive transportation in continental waters did not left any stigmata; 2) or that post-depositional alteration in the site erased the stigmata of this post-marine transportation; 3) or that humans collected them directly in marine beaches (not yet localized) or in slightly derived deposits (still to be identified).

References

1. Biberson, P. *Le Paléolithique inférieur du Maroc atlantique* (Publications du Service des Antiquités du Maroc 17, 1961).

2. Texier, J.-P., Lefèvre, D., Raynal, J.-P. & El Graoui, M. Lithostratigraphy of the littoral deposits of the last one million years in the Casablanca region (Maroc). *Quaternaire* **13**, 23-41 (2002).

3. Cailleux, Y. Le Cambrien et l'Ordovicien du Maroc central méridional. *Bull. Inst. Sci. de Rabat* **18**, 10-31 (1994).

4. Delarue, J., Destombes, J. & Jeannette, A. Etude géotechnique de la région de Casablanca. *Notes* et *Mémoires du Service géologique du Maroc* **130**, 53-143 (1956).

5. Destombes, J. & Jeannette, A. Etude géotechnique de la région de Casablanca (géologie, matières premières minérales, sols). *Notes et mémoires du Service géologique du Maroc* **130**, Editions du Service géologique du Maroc, Rabat, 178 p., carte h.t. (1956).

6. Destombes, J. & Jeannette, A. Mémoire explicatif de la carte géotechnique de la Meseta côtière à l'Est de Casablanca au 1/50 000. Régions de Mohammedia, Bouznika et Ben-Slimane. *Notes et mémoires du Service géologique du Maroc* **180 bis**, Editions du Service géologique du Maroc, Rabat, 104 p., pl. h.t., carte h.t. (1966).

7. André, P.J., Boissin, J.P., Corsini, M. & Renard, J.P. Sur le Cambrien de la région de Casablanca (Maroc) : la série de Dar Bou Azza. *Bull. Soc. Geol. Fr.* **6**, l161-1170 (1987).

8. Hamoumi, N. & Izart, A. Le Cambro-Ordovicien de la Méséta Côtière et le Dévono-Carbonifère du Bassin de Sidi Bettache. *Bull. Inst. Sci. de Rabat* **9**, 117 (1984).

9. Zahraoui, M. La plate-forme carbonatée dévonienne du Maroc occidental et sa dislocation hercynienne. PhD Dissertation, Brest (1991).

10. Hamoumi, N. Le Paléozoïque inférieur de la Méséta Côtière entre Casa et Rabat in *Livret guide : géotraversée du Maroc hercynien (zone Nord)*. Excursion B1, P.I.C.G. 27, Y. Cailleux coord., Ministère Energie et Mines, Rabat (1983).

11. Hamoumi, N. La plateforme ordovicienne du Maroc : dynamique des ensembles sédimentaires. PhD Dissertation, Strasbourg (1988).

12. Lecointre, G. & Gigout, M. Carte géologique provisoire des environs de Casablanca au 1/200 000. Notice explicative. *Notes et mémoires du Service géologique du Maroc* **72 bis**, Rabat, Imprimerie Officielle, 42 p. (1950).

13. Destombes, J. & Jeannette, A. Etude géotechnique de la région de Casablanca (géologie, matières premières minérales, sols). *Notes et mémoires du Service géologique du Maroc* **130**, Editions du Service géologique du Maroc, Rabat, 178 p., carte h.t. (1956).

14. Fernandes, P. Itinéraires et transformations du silex : une pétroarchéologie refondée, application au Paléolithique moyen. PhD Dissertation, Bordeaux (2012).

15. Fernandes, P., Raynal, J.-P. & Mohib, A. L'exploitation des ressources minérales par les hommes du Second Acheuléen régional (Grottes des Rhinocéros, Casablanca, Maroc) in *Préhistoire de Casablanca. I - La Grotte des Rhinocéros (fouilles 1991 et 1996)* (eds. Raynal, J.-P. & Mohib, A.) 145-154 (Institut National des Sciences de l'Archéologie et du Patrimoine, VESAM vol. VI, 2016).

16. Fernandes, P. *et al.* Origins of prehistoric flints: The neocortex memory revealed by scanning electron microscopy. *C. R. Palevol* **6**, 557-568 (2007).

17. Thiry, M., Fernandes, P., Milnes, A. & Raynal, J.-P. Driving forces for the weathering and alteration of silica in the regolith: implications for studies of prehistoric flint tools. *Earth-Sci. Rev.* **136**, 141-154 (2014).

18. Delvigne, V. *et al*. Barremian–Bedoulian flint humanly transported from the west bank of the Rhône to the Massif-Central Highlands–A diachronic perspective. *C. R. Palevol* **18**, 90-112 (2019).

19. Delvigne, *et al*. Grand-Pressigny was not alone: acquiring and sharing data about raw materials in the collective research project "Réseau de lithothèques en region Centre-Val de Loire" (France). *J. Lithic Studies* **5**, https://doi.org/10.2218/jls.2798.

20. Roch, E. Histoire stratigraphique du Maroc. *Notes et mémoires du Service géologique du Maroc* **80**, Toulouse, 435 p., XXII pl. h.t. (1950).

21. Gigout, M. Etudes géologiques sur la Méséta marocaine occidentale (arrière-pays de Casablanca, Mazagan et Safi). *Notes et mémoires du Service géologique du Maroc* **86**, Rabat, Imprimerie Maroc-Matin, tome I: texte, 507 p. (1951).

22. Salvan, H.M. Les terrains de recouvrement des séries phosphatées de Khouribga (Maroc) et les problèmes qu'ils soulèvent. *Notes et Mémoires du Service Géologique du Maroc* **t. 1, 71**, 37-40 (1948).

23. Salvan, H.M. Étude préliminaire du gisement des Meskala. Unpublished report Service Études des gisements OCP, 5 (1963).

24. Salvan, H.M. Géologie des gîtes minéraux marocains, *Notes et Mémoires du Service Géologique du Maroc*, **t. 3, 276**, 392 p. (1986).

25. Termier, H. *Etudes géologiques sur le Maroc central et le Moyen Atlas septentrional. Tome I : Les terrains primaires et le Permo-Trias. Tome II : Les terrains post-triasiques. Tome III : Paléontologie, Pétrographie. Tome IV : Atlas des figures et des tableaux hors-texte* (Protectorat de la République française au Maroc, Direction générale des Travaux Publics, Service des Mines et de la Carte Géologique, Notes et Mémoires n° 33, 1566 p., 1936).

26. Bouzouggar, A. Matières premières, processus de fabrication et de gestion des supports d'outils dans la séquence atérienne de la grotte d'El Mnasra I (ancienne grotte des Contrebandiers) à Témara (Maroc). PhD dissertation, Bordeaux (1997).

27. El Amrani El Hassani, I.E. & Morala, A. Réflexions méthodologiques sur la lithologie des assemblages paléolithiques de la région de Rabat-Témara (Maroc*). Bulletin de l'Institut Scientifique de Rabat* **34**, 1-14 (2012).

28. Morala, A. & El Amrani El Hassani, I.E. Lithologie in *Préhistoire de la région de Rabat-Tamara* (eds. El Hajraoui, M.-A., Nespoulet, R., Debénath, A. & Dibble, H.L.) 249-252 (VESAM, 2012).

29. Morala, A., El Amrani El Hassani, I.E. & Debenath, A. Lithologie des sites de la région de Rabat-Témara in *Préhistoire de la région de Rabat-Tamara* (eds. El Hajraoui, M.-A., Nespoulet, R., Debénath, A. & Dibble, H.L.) 82-86 (VESAM, 2012).

30. Morala, A., El Amrani El Hassani, I.E. & Debenath, A. Lithologie : données générales. in *Préhistoire de la région de Rabat-Tamara* (eds. El Hajraoui, M.-A., Nespoulet, R., Debénath, A. & Dibble, H.L.) 154-164 (VESAM, 2012).

31. Niftah, S., Debenath, A. & Miskovsky, J.-C. Origine du remplissage sédimentaire des grottes de Témara (Maroc) d'après l'étude des minéraux lourds et l'étude exoscopique des grains de quartz. *Quaternaire* **16**, 73-83 (2005).

3. Supplementary Information accompanying the section "Flint knapping activities"

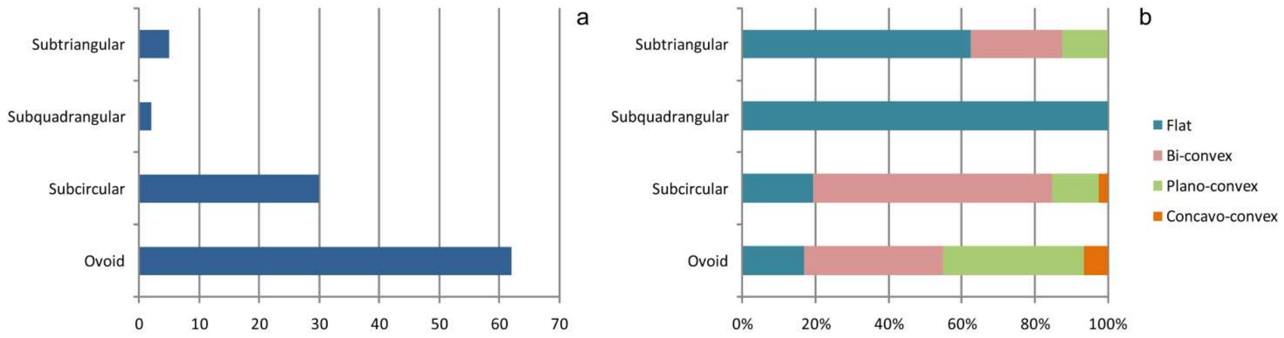

Figure S5. a: shape distribution of the flint pebbles; b: frequency of the flint pebble cross-sections, grouped by pebble shape.

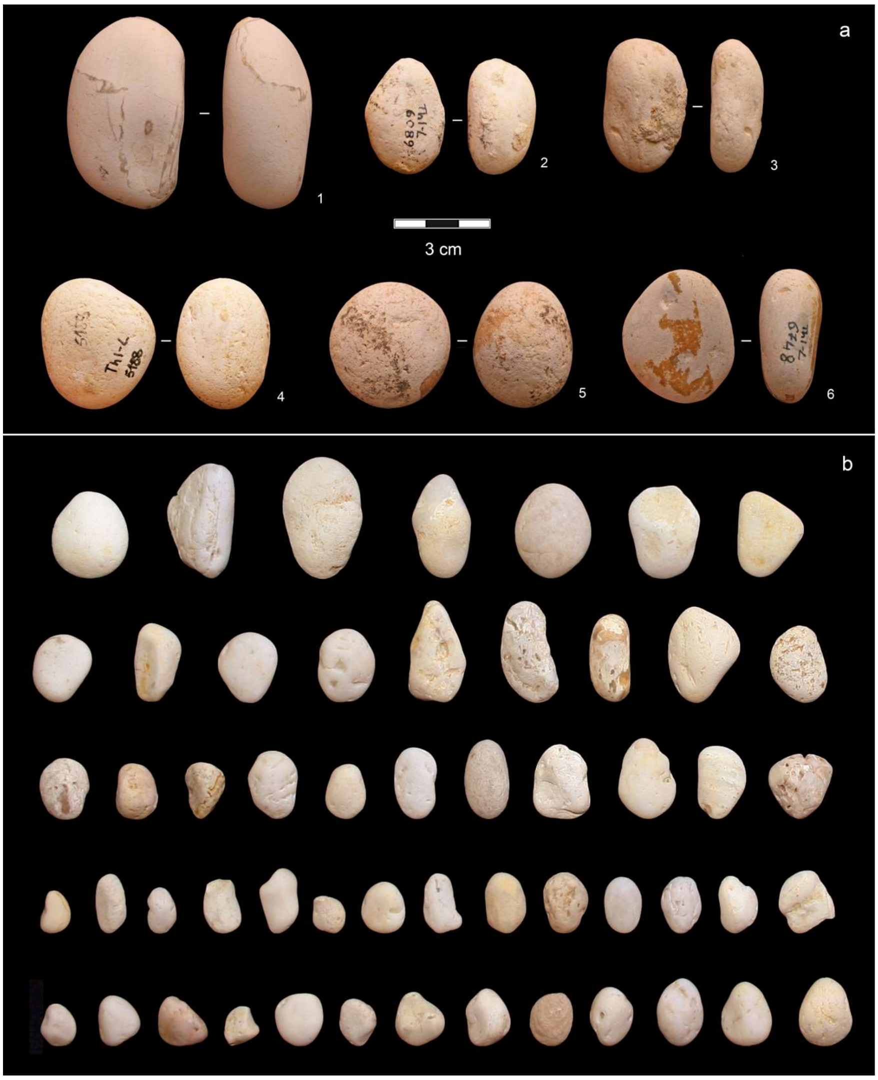

Figure S6. a: most frequent morphologies of the flint pebbles. These pebbles have been coordinated in three dimensions during excavations. 1-3: ovoid plano-convex pebbles; 4,5: subcircular bi-convex pebbles; 6: subcircular flat pebble; b: an example of the different dimensions of the flint pebbles recovered from the sieving of the archaeological deposit (photos by R. Gallotti).

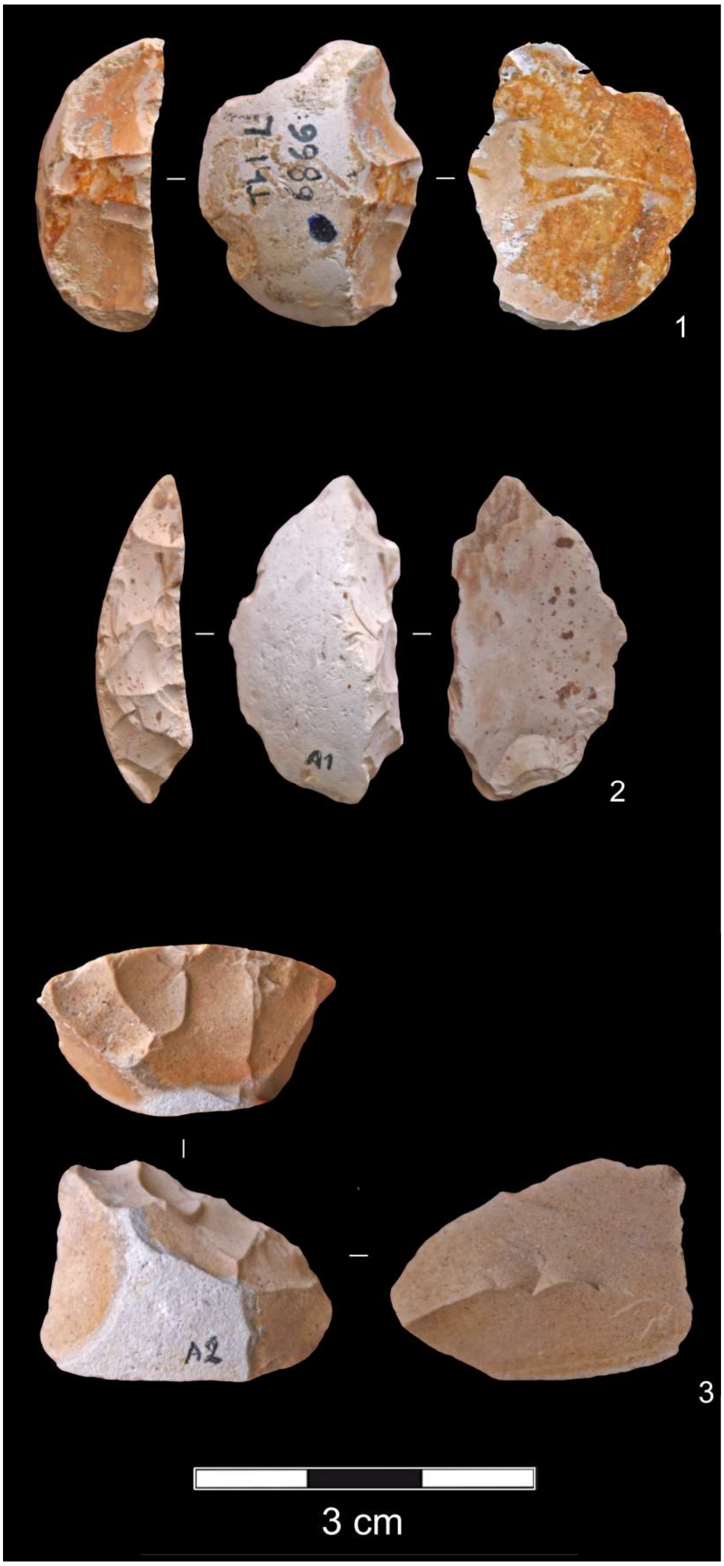

Figure S7. Retouched flint flakes (photo by R. Gallotti).

Pebbles with percussion marks

6 pebbles bear percussion marks. Battering impact damages are located on the face of subcircular flat pebbles, near the distal edge in two instances (Fig. S8: 1,2). The first one shows a flat cross-section and its dimensions are 35x34x18 mm; the second is a bi-convex pebble whose dimensions are 47x46x30 mm. In another case, the battering marks are associated with a removal bearing a central impact point and are located on the right edge. This is an ovoid bi-convex pebble of 34x27x21 mm (Fig. S8: 6). These 3 pieces could be potential hammerstones for a full flaking stage of the bipolar-on-anvil percussion.

The other 3 pebbles show pitted areas on one extremity of the long axis (Fig. S8: 3-5). They are ovoid pebbles with a plano-convex or bi-convex cross-section, very similar in their dimensions (42x32x23 mm; 48x39x23 mm; 47x39x30 mm). They could be used as hammerstones. However, the presence of a small removal on the opposite extremity of one of them (Fig. S8: 3) could indicate that they were actually pebbles that the knappers had tried to split along the longitudinal axis.

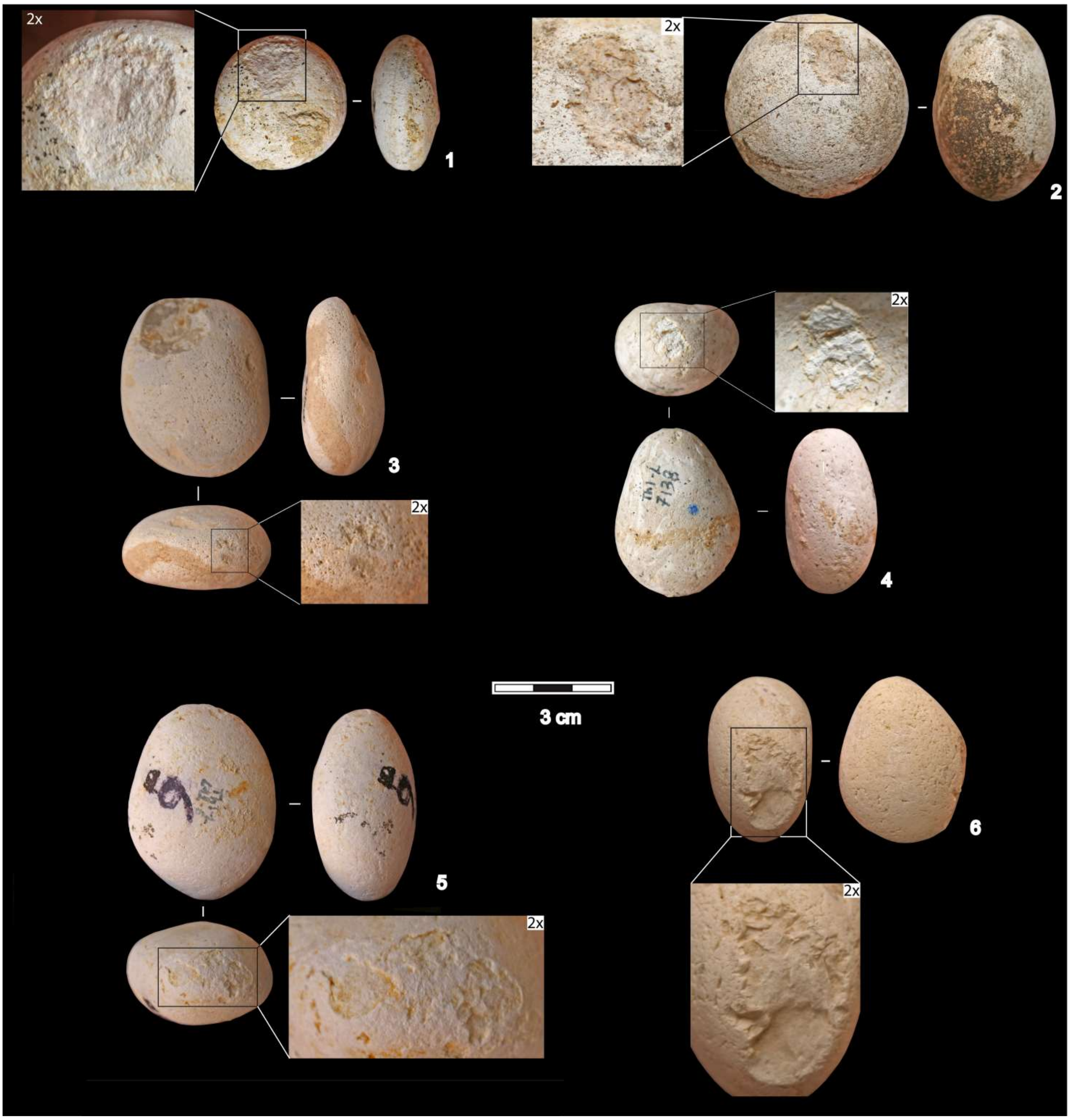

Figure S8. Flint pebbles with percussion marks (photo by R. Gallotti).
